# Molecular and histological correlates of cognitive decline across age in male C57BL/6J mice

**DOI:** 10.1002/brb3.2736

**Published:** 2022-08-15

**Authors:** Rachel Britton, Angela T. Liu, Sanket V. Rege, Julia M. Adams, Lily Akrapongpisak, David Le, Raniel Alcantara‐Lee, Raul A. Estrada, Rebecca Ray, Sara Ahadi, Ian Gallager, Cindy F. Yang, S. Sakura Minami, Steven P. Braithwaite, Eva Czirr, Meghan Kerrisk Campbell

**Affiliations:** ^1^ Alkahest, Inc. San Carlos California USA; ^2^ Coda Biotherapeutics South San Francisco California USA; ^3^ University of Queensland Herston Queensland Australia; ^4^ Fountain Therapeutics South San Francisco California USA; ^5^ 202 Chives Way Walnut Creek California USA; ^6^ Confluence Therapeutics South San Francisco California USA

**Keywords:** aging, behavior, blood‐brain barrier, hippocampus, neuroinflammation, T lymphocytes

## Abstract

**Introduction:**

Increasing age is the number one risk factor for developing cognitive decline and neurodegenerative disease. Aged humans and mice exhibit numerous molecular changes that contribute to a decline in cognitive function and increased risk of developing age‐associated diseases. Here, we characterize multiple age‐associated changes in male C57BL/6J mice to understand the translational utility of mouse aging.

**Methods:**

Male C57BL/6J mice from various ages between 2 and 24 months of age were used to assess behavioral, as well as, histological and molecular changes across three modalities: neuronal, microgliosis/neuroinflammation, and the neurovascular unit (NVU). Additionally, a cohort of 4‐ and 22‐month‐old mice was used to assess blood‐brain barrier (BBB) breakdown. Mice in this cohort were treated with a high, acute dose of lipopolysaccharide (LPS, 10 mg/kg) or saline control 6 h prior to sacrifice followed by tail vein injection of 0.4 kDa sodium fluorescein (100 mg/kg) 2 h later.

**Results:**

Aged mice showed a decline in cognitive and motor abilities alongside decreased neurogenesis, proliferation, and synapse density. Further, neuroinflammation and circulating proinflammatory cytokines were increased in aged mice. Additionally, we found changes at the BBB, including increased T cell infiltration in multiple brain regions and an exacerbation in BBB leakiness following chemical insult with age. There were also a number of readouts that were unchanged with age and have limited utility as markers of aging in male C57BL/6J mice.

**Conclusions:**

Here we propose that these changes may be used as molecular and histological readouts that correspond to aging‐related behavioral decline. These comprehensive findings, in the context of the published literature, are an important resource toward deepening our understanding of normal aging and provide an important tool for studying aging in mice.

## Graphical abstract

We identify robust molecular and histological changes with age in male C57BL/6J mice that may be used as correlates of aging‐related cognitive decline. These comprehensive findings, in the context of the published literature, are an important resource towards deepening our understanding of normal aging and provide an important tool for studying aging in mice.

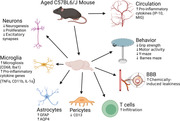

## 1 INTRODUCTION

Aging is associated with a progressive decline in numerous functions and an increased incidence of frailty and disease (Heinze‐Milne et al., 2019; Hou et al., 2019; Kane et al., 2019; Lopez‐Otinet al., 2013). Specifically in the aged brain, there is a loss of synaptic connections (Morrison & Baxter, 2012), increased neurodegeneration (Wyss‐Coray, 2016), heightened neuroinflammatory responses (Spencer et al., 2017) from both microglia (Niraulaet al., 2017) and astrocytes (Boisvert et al., 2018), a greater number of infiltrating macrophages from the periphery (Scheiblich et al., 2020), vascular dysfunction (Ungvari et al., 2018), loss of blood‐brain barrier (BBB) integrity (Benveniste et al., 2018; Kress et al., 2014), and degeneration of the auditory system (Kobrina et al., 2020), which can each contribute to a decline in cognitive function (Bettio et al., 2017; Weber et al., 2015). Research into aging‐related mechanisms has expanded rapidly over the past few years leading to many potential therapeutics to treat aging‐related diseases in humans (Bakula et al., 2019; Hodgson et al., 2020). Studying behavior in aged mice as a model for human cognitive decline is necessary but remains challenging. Behavioral protocols need to be optimized for each age, strain, and animal source (Ryman & Lamb, 2006; Scearce‐Levie, 2011; Sukoff Rizzo et al., 2018; Sukoff Rizzo & Silverman, 2016), which is time‐consuming and often requires specialized equipment. Additionally, aged animals are sensitive to environmental changes, and behavioral readouts can be variable within and between different cohorts and experimenters. Furthermore, interpreting cognitive decline in aged mice is complicated by the fact that aged animals also have motor impairments, so the readouts for many cognitive tasks are influenced by both cognition and ambulation. Here we aim to form a comprehensive profile of the molecular and histological changes that are robustly modulated with aging in male C57BL/6J mice, which is the most common inbred mouse strain used in the neuroscience field. These endpoints are typically more straightforward to implement and do not suffer from the same variability issues as behavior. We propose that histological and molecular changes therefore may provide more granularity and be more consistent biomarkers of aging. While we will not opine on which is more functionally relevant than the other, we focus on three modalities: neuronal, microgliosis/neuroinflammation, and the neurovascular unit (NVU).

## 2 MATERIALS AND METHODS

### 2.1 Animals

All animal handling and use was in accordance with Institutional Animal Care and Use Committee approved standard guidelines, protocol ALK‐005. Male C57BL/6J mice were ordered from Jackson Laboratory (Sacramento, CA) and shipped to Alkahest prior to the start of each study. All animals were acclimated in house for at least 2 weeks prior to the start of the experiments. Upon arrival, all mice were housed with a unique identification number at standard temperature (22 ± 1°C) and in a light‐controlled environment (lights on from 7 am to 7 pm) with ad libitum access to food and water.

**Table:** 

**Cohort**	**Young Age**	**Old Age**	**Other Ages Used**	**Figures**
Cohort 1	3 months	20 months	–	Figure 1A (Y‐maze)
Cohort 2	2 months	22 months	–	Figure 1A (Y‐maze), 1B (Barnes maze)
Cohort 3	3 months	20 months	–	Figure 1C,D (Y‐maze)
Cohort 4	6.5 months	22.5 months	–	Figure 1E,F (Grip strength)
Cohort 5	3 months	24 months	6, 12, 18 months	Figure 2A,B (DCX)
Cohort 6	2 months	24 months	–	Figure 2C,D (Ki67), Supplementary Figure 1B,1C (BrdU)
Cohort 7	3 months	24 months	12, 18 months	Figure 2E,F (Synapses); Figure 3 (Microglia); Figure 4D–G (GFAP); Figure 5 (T cells); Supplementary Figure 1A, 1D–F (Gene expression); Supplementary Figure 2 (Microglia/Inflammation); Supplementary Figure 3 (Gene expression)
Cohort 8	3 months	22.5 months	–	Figure 4A–C (GFAP), 4H–J (Western blots)
Cohort 9	4 months	22 months	–	Figure 6 (LPS)

**FIGURE 1:**
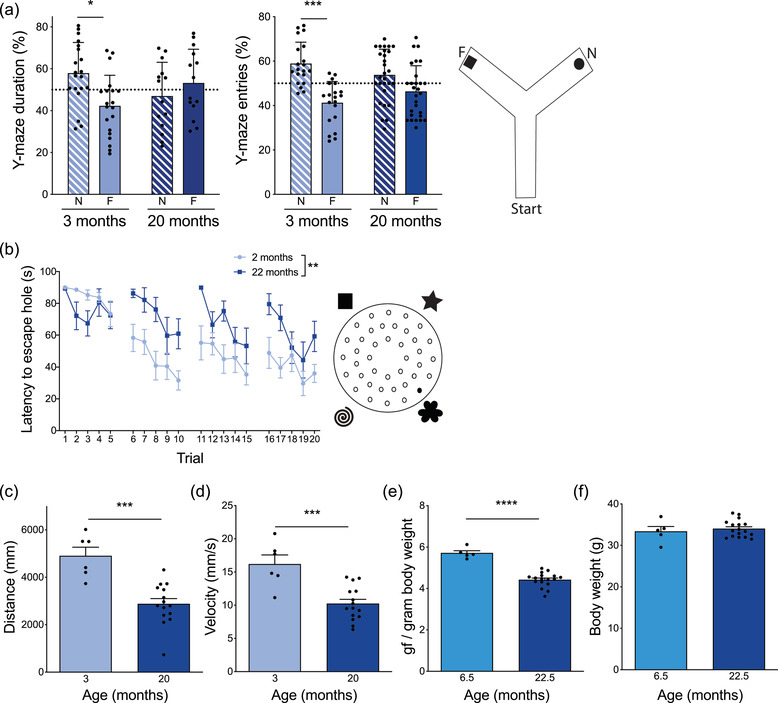
Impaired cognitive and motor function in aged mice. (A) Average percent duration spent in the novel (N) and familiar (F) arms of Y‐maze during the testing phase for young (3 month) and aged (20 month) mice. *n* = 20–28 mice per group. Three‐way repeated measures ANOVA: Arm x Age x Experiment *F* = 9.162, *p* = 0.0032, followed by Wilcoxon matched‐paired signed rank tests: 3 month *p = 0.0215, 20 month *p* = 0.7282. Average percent entries into the novel (N) and familiar (F) arms of Y‐maze during the testing phase for young (3 month) and aged (20 month) mice. *n* = 20–28 mice per group. Three‐way repeated measures ANOVA: Arm x Age x Experiment *F* = 4.994, *p* = 0.0071, followed by Wilcoxon matched‐paired signed rank tests: 3 month ***p = 0.0004, 20 month *p* = 0.0863. Cartoon depicting Y‐maze set up. (B) Average latency to find escape hole in Barnes maze task over the course of 4 days with 5 trials per day in young (2 month) and aged (22 month) mice. *n* = 8–10 mice per group. Mixed‐effects analysis with repeated measures: Trial x Age F = 2.401, *p* = 0.0011; Trial *F* = 7.394, *p*<0.0001; Age *F* = 14.21, **p = 0.0017. Cartoon depicting Barnes maze set up. (C) Total distance traveled in 5 min during training phase of Y‐maze by young (3 month) and aged (20 month) mice. *n* = 6–15 mice per group. Mann–Whitney test ***p = 0.0001. (D) Average velocity over 5 min during training phase of Y‐maze of young (3 month) and aged (20 month) mice. *n* = 6–15 mice per group. Mann–Whitney test ***p = 0.0007. (E) Average maximum grip strength across 4 trials (gf, gram‐force) normalized to individual mouse body weight of young (6.5 month) and aged (22.5 month) mice. *n* = 5–17 mice per group. Mixed‐effects analysis with repeated measures: Trial x Age *F* = 3.986, *p* = 0.0118; Trial F = 2.008, *p* = 0.1389; Age *F* = 31.7, *p*<0.0001, followed by Mann–Whitney test ****p<0.0001. F. Average body weight of young (6.5 month) and aged (22.5 month) mice. *n* = 5–17 mice per group. Mann–Whitney test *p* = 0.8201. All data are shown as mean ± s.e.m. Abbreviations: ANOVA, analysis of variance

**FIGURE 2:**
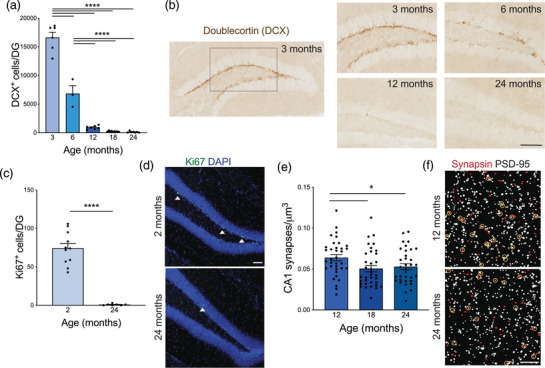
Reduced neurogenesis, proliferation, and synaptic density in aged mice. (A) Number of Doublecortin‐positive (DCX+) cells per dentate gyrus (DG) as a marker of newborn neurons in mice 3–24 months of age. *n* = 3–9 mice per group. Nested one‐way ANOVA *F* = 221.9, *p*<0.0001, followed by Tukey's multiple comparisons test: 3 vs. 6 ****p<0.0001, 3 vs. 12 ****p<0.0001, 3 vs. 18 ****p<0.0001, 3 vs. 24 ****p<0.0001, 6 vs. 12 ****p<0.0001, 6 vs. 18 ****p<0.0001, 6 vs. 24 ****p<0.0001, 12 vs. 18 *p* = 0.7895, 12 vs. 24 *p* = 0.7343, 18 vs. 24 *p* = 0.9998. (B) Representative images of DCX staining in the DG of mice 3–24 months of age. Scale bar 100 mm. (C) Number of Ki67‐positive cells per DG as a marker of cell proliferation in young (2 month) and aged (24 month) mice. *n* = 7–11 mice per group. Nested *t*‐test *F* = 82.56, ****p<0.0001. (D) Representative images of Ki67 (green) and nuclear DAPI (blue) staining in the DG of young (2 month) and aged (24 month) mice. Ki67‐positive cells are indicated with white arrow heads. Scale bar 100 mm. (E). Number of juxtaposed Synapsin and PSD‐95 puncta per μm^3^ in the CA1 region of the hippocampus as a readout for excitatory synapse density in adult (12 month), middle‐aged (18 month), and aged (24 month) mice. *n* = 34–35 images from 6 mice per group. One way ANOVA F = 3.623, *p* = 0.0302, followed by unpaired *t*‐tests: 12 vs. 18 *p = 0.0154, 12 vs. 24 *p = 0.04, 18 vs. 24 *p* = 0.6518. (F) Representative images of a single z‐plane of thresholded Synapsin (red) and PSD‐95 (white) with juxtaposed synapses circled in yellow in the CA1 of adult (12 month) and aged (24 month) mice. Scale bar 5 mm. All data are shown as mean ± s.e.m. Abbreviations: DCX, doublecortin; DG, dentate gyrus; PSD‐95, post‐synaptic density protein 95; CA1, *Cornu Ammonis* region 1; ANOVA, analysis of variance

**FIGURE 3:**
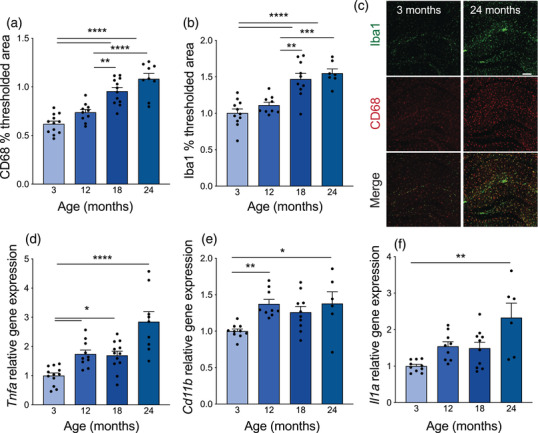
Stepwise increase in hippocampal microgliosis and elevated proinflammatory cytokines with age. (A) Average thresholded percent area of CD68‐positive microglia in the hippocampus of mice 3–24 months of age. *n* = 9–12 mice per group. Nested one‐way ANOVA *F* = 28.96, *p*<0.0001, followed by Tukey's multiple comparisons test: 3 vs. 12 *p* = 0.1376, 3 vs. 18 ****p<0.0001, 3 vs. 24 ****p<0.0001, 12 vs. 18 **p = 0.0014, 12 vs. 24 ****p<0.0001, 18 vs. 24 *p* = 0.1216. (B) Average thresholded percent area of Iba1‐positive microglia in the hippocampus of mice 3–24 months of age. *n* = 7–11 mice per group. Nested one‐way ANOVA *F* = 17.94, *p*<0.0001, followed by Tukey's multiple comparisons test: 3 vs. 12 *p* = 0.5851, 3 vs. 18 **** *p*<0.0001, 3 vs. 24 **** *p*<0.0001, 12 vs. 18 **p = 0.0014, 12 vs. 24 ***p = 0.0004, 18 vs. 24 *p* = 0.8235. (C) Representative images from hippocampus of 3‐ and 24‐month‐old mice of Iba1 (green) and CD68 (red) microglia. Scale bar 100 mm. (D) Average hippocampal *Tnfa* gene expression relative to *Gapdh* measured by TaqMan qPCR in mice 3–24 months of age. *n* = 9–12 mice per group. Kruskal–Wallis test *p*<0.0001, followed by Dunn's multiple comparisons test: 3 vs. 12 *p = 0.0188, 3 vs. 18 *p = 0.0206, 3 vs. 24 ****p<0.0001, 12 vs. 18 *p*>0.9999, 12 vs. 24 *p* = 0.2929, 18 vs. 24 *p* = 0.1616. (E) Average hippocampal *Cd11b* gene expression relative to *Gapdh* measured by SYBR qPCR in mice 3–24 months of age. *n* = 6–10 mice per group. Kruskal–Wallis test *p* = 0.0024, followed by Dunn's multiple comparisons test: 3 vs. 12 **p = 0.0040, 3 vs. 18 *p* = 0.0983, 3 vs. 24 *p = 0.0217, 12 vs. 18 *p*>0.9999, 12 vs. 24 *p*>0.9999, 18 vs. 24 *p*>0.9999. (F) Average hippocampal *Il1a* gene expression relative to *Gapdh* measured by SYBR qPCR in mice 3–24 months of age. *n* = 6–10 mice per group. Kruskal–Wallis test *p* = 0.0033, followed by Dunn's multiple comparisons test: 3 vs. 12 *p* = 0.0788, 3 vs. 18 *p* = 0.2415, 3 vs. 24 **p = 0.0024, 12 vs. 18 *p*>0.9999, 12 vs. 24 *p*>0.9999, 18 vs. 24 *p* = 0.4666. All data are shown as mean ± s.e.m. Abbreviations: ANOVA, analysis of variance; Iba1, ionized calcium‐binding adapter molecule 1; *Gapdh*, glyceraldehyde‐3‐phosphate dehydrogenase; qPCR, quantitative polymerase chain reaction; *Tnfa*, tumor necrosis factor alpha; *Il1a*, interleukin 1 alpha

**FIGURE 4:**
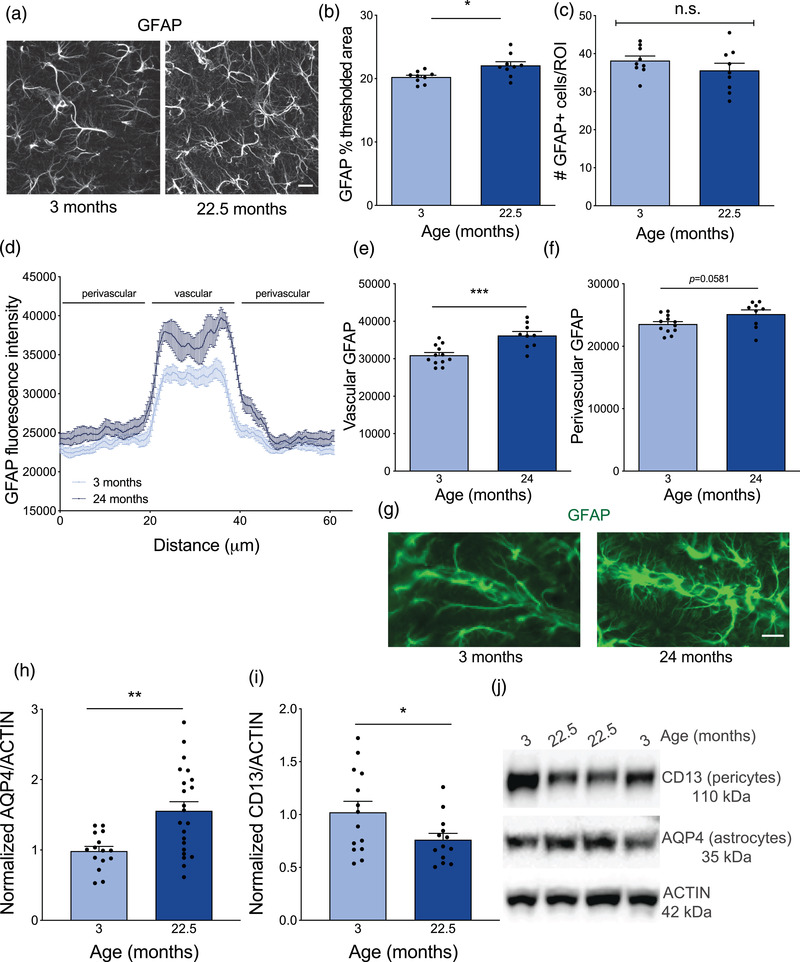
Increase in astrocyte reactivity and reduction in pericytes at the neurovascular unit with age. (A) Representative maximum intensity projections of astrocyte marker GFAP in the CA1 region of the hippocampus in young (3 month) and aged (22.5 month) mice. Scale bar 30 mm. (B) Average thresholded percent area of GFAP in the CA1 region of the hippocampus in young (3 month) and aged (22.5 month) mice. *n* = 9 mice per group. Nested *t*‐test *p = 0.0145. (C) Average number of GFAP positive cells in a region of interest (ROI) in the CA1 region of the hippocampus in young (3 month) and aged (22.5 month) mice. *n* = 9 mice per group. Nested *t*‐test *p* = 0.2719. (D) Cross‐sectional quantification of GFAP fluorescent intensity across large descending vessels in the CA1 region of the hippocampus of young (3 month) and aged (24 month) mice. *n* = 9–12 mice per group. (E) Average fluorescence intensity of GFAP in the vascular region across large descending vessels of the CA1 region of the hippocampus of young (3 month) and aged (24 month) mice. *n* = 9–12 mice per group. Nested *t*‐test ***p = 0.0007. (F) Average fluorescence intensity of GFAP in the perivascular region surrounding the large descending vessels of the CA1 region of the hippocampus of young (3 month) and aged (24 month) mice. *n* = 9–12 mice per group. Nested *t*‐test *p* = 0.0581. (G). Representative images of GFAP (green) staining surrounding large descending vessels in the CA1 region of the hippocampus of young (3 month) and aged (24 month) mice. Scale bar 20 mm. (H) Average relative protein expression of astrocyte endfoot protein AQP4 in cortical lysates of young (3 month) and aged (22.5 month) mice measured by western blot and normalized to ACTIN loading control. *n* = 16–22 mice per group. Mann–Whitney test **p = 0.0033. (I). Average relative protein expression of pericyte marker CD13 in cortical lysates of young (3 month) and aged (22.5 month) mice measured by western blot and normalized to ACTIN loading control. *n* = 13–14 mice per group. Unpaired *t*‐test *p = 0.0478. (J). Representative western blot bands of AQP4, CD13, and ACTIN. All data are shown as mean ± s.e.m. Abbreviations: GFAP, glial fibrillary acidic protein; ROI, region of interest; AQP4, aquaporin‐4; CA1, *Cornu Ammonis* region 1

**FIGURE 5:**
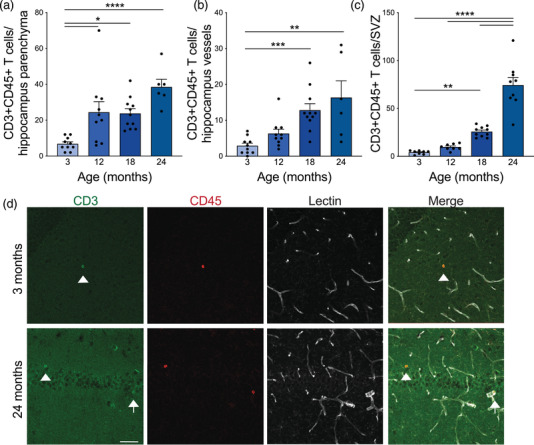
Stepwise increase in T cell infiltration into the hippocampus and subventricular zone (SVZ) with age. (A) Number of CD3–CD45‐double positive, LECTIN‐negative T cells counted in the parenchyma of the hippocampus of mice 3–24 months of age. *n* = 6–11 mice per group. Nested one‐way ANOVA *F* = 9.554, *p* = 0.0001, followed by Tukey's multiple comparisons test: 3 vs. 12 *p = 0.0113, 3 vs. 18 *p = 0.0129, 3 vs. 24 ****p<0.0001, 12 vs. 18 *p* = 0.9992, 12 vs. 24 *p* = 0.1181, 18 vs. 24 *p* = 0.0865. (B) Number of CD3–CD45‐double positive T cells counted in the LECTIN‐positive blood vessels of the hippocampus of mice 3–24 months of age. *n* = 6–11 mice per group. Kruskal–Wallis test *p* = 0.0003, followed by Dunn's multiple comparisons test: 3 vs. 12 *p* = 0.5355, 3 vs. 18 ***p = 0.0006, 3 vs. 24 **p = 0.0047, 12 vs. 18 *p* = 0.1893, 12 vs. 24 *p* = 0.3570, 18 vs. 24 *p*>0.9999. (C) Number of CD3‐CD45‐double positive T cells counted in the SVZ of mice 3–24 months of age. *n* = 8–10 mice per group. Nested one‐way ANOVA *F* = 53.54, *p*<0.0001, followed by Tukey's multiple comparisons test: 3 vs. 12 *p* = 0.8266, 3 vs. 18 **p = 0.0066, 3 vs. 24 ****p<0.0001, 12 vs. 18 *p* = 0.0589, 12 vs. **** 24 *p*<0.0001, 18 vs. **** 24 *p*<0.0001. (D) Representative images of CD3 (green), CD45 (red), and LECTIN (white) staining in the hippocampus of young (3 month) and aged (24 month) mice. White arrowheads identify CD3–CD45‐double positive T cells in the hippocampal parenchyma and white arrows identify cells in the LECTIN‐positive blood vessels. Scale bar 50 mm. All data are shown as mean ± s.e.m. Abbreviations: SVZ, subventricular zone; ANOVA, analysis of variance

**FIGURE 6:**
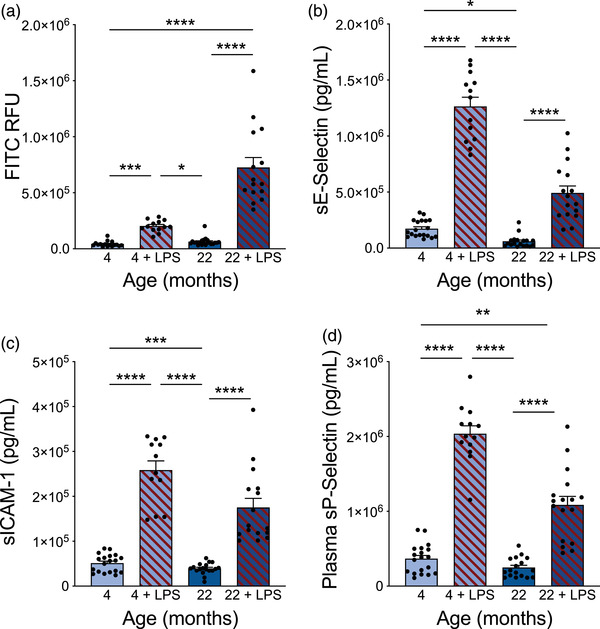
High‐dose LPS caused BBB leakiness and increased plasma levels of soluble CAMs. (A) Sodium fluorescein (0.4 kDa) leakiness into the brain of young (4 month) and aged (22 month) mice with and without a high, acute dose of LPS (10 mg/kg). *n* = 13–21 mice per group. Kruskal–Wallis test *p*<0.0001, followed by Dunn's multiple comparisons test: 4 vs. 4+LPS ***p = 0.0001, 4 vs. 22 *p* = 0.5342, 4 vs. 22+LPS ****p<0.0001, 4+LPS vs. 22 *p = 0.0213, 4+LPS vs. 22+LPS *p* = 0.2418, 22 vs. 22+LPS ****p<0.0001. (B) Soluble E‐Selectin levels in plasma of young (4 month) and aged (22 month) mice with and without LPS treatment. *n* = 13–19 mice per group. Kruskal–Wallis test *p*<0.0001, followed by Dunn's multiple comparisons test: 4 vs. 4+LPS ****p<0.0001, 4 vs. 22 *p = 0.0393, 4 vs. 22+LPS *p* = 0.0736, 4+LPS vs. 22 ****p<0.0001, 4+LPS vs. 22+LPS *p* = 0.1966, 22 vs. 22+LPS ****p<0.0001. (C) Soluble ICAM‐1 levels in plasma of young (4 month) and aged (22 month) mice with and without LPS treatment. *n* = 12–19 mice per group. Kruskal–Wallis test *p*<0.0001, followed by Dunn's multiple comparisons test: 4 vs. 4+LPS ****p<0.0001, 4 vs. 22 *p*>0.9999, 4 vs. 22+LPS ***p = 0.0002, 4+LPS vs. 22 ****p<0.0001, 4+LPS vs. 22+LPS *p*>0.9999, 22 vs. 22+LPS ****p<0.0001. (D) Soluble P‐Selectin levels in plasma of young (4 month) and aged (22 month) mice with and without LPS treatment. *n* = 12–19 mice per group. Kruskal–Wallis test *p*<0.0001, followed by Dunn's multiple comparisons test: 4 vs. 4+LPS ****p<0.0001, 4 vs. 22 *p*>0.9999, 4 vs. 22+LPS **p = 0.0041, 4+LPS vs. 22 ****p<0.0001, 4+LPS vs. 22+LPS *p*>0.9999, 22 vs. 22+LPS ****p<0.0001. All data are shown as mean ± s.e.m. Abbreviations: LPS, lipopolysaccharide; BBB, blood‐brain barrier; CAM, cell adhesion molecule; FITC, fluorescein isothiocyanate; RFU, relative fluorescent units; ANOVA, analysis of variance; sICAM‐1, soluble intercellular adhesion molecule‐1

To minimize the number of animals used per experiment, brains from cohorts 5–8 were sub‐dissected and collected for 3 separate techniques. One hemibrain was used for histology, while the other hemibrain was further dissected into hippocampus, used for qPCR, and cortex, used for western blots.

Cohort 6 was used for proliferation experiments in Figure 2C,D and Supplementary Fig. 1B,C. The 2‐ and 24‐month‐old mice were dosed daily for 7 days IP with saturating amounts of 5‐bromo‐2’‐deoxyuridine (BrdU, B5002‐5G, Sigma Aldrich, St. Louis, MO) for each age. The 2‐month‐old mice were dosed with 500 mg/kg BrdU while the 24‐month‐old mice were dosed with 150 mg/kg BrdU. Mice were then sacrificed 24 h following the last dose of BrdU.

Cohort 9 was used for BBB breakdown experiments as given in Figure 6. The 4‐ and 22‐month‐old mice received 10 mg/kg lipopolysaccharide (LPS serotype O55:B5, L4005, Sigma Aldrich) IP to induce BBB breakdown 6 h prior to sacrifice or saline IP as a control. Additionally, all animals received 100 mg/kg tail vein injection of 0.4 kDa sodium fluorescein (NaF, F6377, Sigma Aldrich) to assess BBB integrity 4 h prior to sacrifice.

### 2.2 Key resources table

**Table:** 

Reagent type (species) or resource	Designation	Source or reference	Identifiers	Additional information
Strain, strain background (Mus musculus)	C57BL/6J	Jackson Laboratory	Stock #: 000664 RRID: IMSR_JAX:000664	Male
Antibody	Anti‐DCX (guinea pig polyclonal)	Millipore	Cat #AB2253 RRID: AB_1586992	IHC 1:2000
Antibody	Anti‐Ki67 (rabbit polyclonal)	Abcam	Cat #ab15580 RRID: AB_443209	IHC 1:500
Antibody	Anti‐BrdU, clone BU1/75 (ICR1) (rat monoclonal)	Abcam	Cat #ab6326 RRID: AB_305426	IHC 1:500
Antibody	Anti‐Synapsin1/2 (chicken polyclonal)	Synaptic Systems	Cat # 106006 RRID: AB_262240	IHC 1:750
Antibody	Anti‐PSD‐95 (rabbit monoclonal)	Cell Signaling Technology	Cat # 3450 RRID: AB_2292883	IHC 1:250
Antibody	Anti‐CD68, clone FA‐11 (rat monoclonal)	Bio‐Rad	Cat #MCA1957 RRID: AB_322219	IHC 1:1000
Antibody	Anti‐Iba1 (rabbit polyclonal)	FUJIFILM Wako Pure Chemical Corporation	Cat #019‐19741 RRID: AB_839504	IHC 1:2500
Antibody	Anti‐GFAP (goat polyclonal)	Abcam	Cat #ab53554 RRID: AB_880202	IHC 1:1000
Antibody	Anti‐CD3, clone 17A2 (rat monoclonal)	BD Biosciences	Cat #555273 RRID: AB_395697	IHC 1:100
Antibody	Anti‐CD45, clone D3F8Q (rabbit monoclonal)	Cell Signaling Technology	Cat #702575 RRID: AB_2799780	IHC 1:200
Antibody	DyLight 594 *Lycopersicon esculentum* (tomato) lectin	Vector Laboratories	Cat #DL‐1177 RRID: AB_2336416	IHC 1:200
Antibody	Anti‐aquaporin‐4 (rabbit polyclonal)	Millipore	Cat #ABN910 RRID: AB_2922395	WB: 1:500
Antibody	Anti‐mouse aminopeptidase N/CD13 (goat polyclonal)	R&D Systems	Cat #AF2335 RRID: AB_2227288	WB 1:500
Antibody	Anti‐actin, HRP conjugated (rabbit monoclonal)	Cell Signaling Technology	Cat #13E5 5125 RRID: AB_1903890	WB 1:5000
Antibody	Alexa 555 or 647 secondaries	Invitrogen		IHC 1:300
Antibody	Biotinylated anti‐guinea pig IgG (goat polyclonal)	Vector Laboratories	Cat #BA‐7000 RRID: AB_2336132	IHC 1:300
Antibody	Anti‐rabbit IgG (H+L), HRP conjugated (donkey polyclonal)	Fisher Scientific	Cat # A16035 RRID: AB_2534709	WB 1:5000
Antibody	Anti‐goat IgG (H+L), HRP conjugated (donkey polyclonal)	Fisher Scientific	Cat #A15999 RRID: AB_2534673	WB 1:5000
Other	Hoechst	Invitrogen	Cat #H3570	IHC 1:10000
Other	Prolong Gold Antifade Mountant	Invitrogen	Cat #P36934	
Chemical compound	3,3′‐Diaminobenzidine tetrahydrochloride (DAB)	Sigma Aldrich	Cat #D5905	
Chemical compound	Citrisolv clearing agent	Decon Labs	Cat #22‐143‐975	
Chemical compound	Cytoseal	Thermo Scientific	Cat #8310‐4	
Chemical compound	RIPA lysis and extraction buffer	Thermo Scientific	Cat #89901	
Chemical compound	Halt protease and phosphatase inhibitor cocktail (100X)	Thermo Scientific	Cat #78446	
Chemical compound	4X Bolt LDS sample buffer	Invitrogen	Cat #B0007	
Other	Bolt 4 to 12%, Bis‐Tris, 1.0 mm, Mini protein gel, 15‐well	Invitrogen	Cat #NW04125BOX	
Commercial assay or kit	Trans‐Blot Turbo Mini 0.2 μm nitrocellulose transfer packs	Bio‐Rad	Cat #1704158	
Chemical compound	Nonfat dry milk, blotting‐grade	Bio‐Rad	Cat #1706404	
Other	PageRuler Plus Prestained Protein Ladder, 10 to 250 kDa	Thermo Scientific	Cat #26619	
Chemical compound	SuperSignal West Pico PLUS Chemiluminescent Substrate	Thermo Scientific	Cat #34580	
Chemical compound, drug	2,2,2‐Tribromoethanol (Avertin)	Sigma Aldrich	Cat #T48402‐25G	1.61g/mL stock diluted 1:40 in sterile saline
Chemical compound, drug	5‐Bromo‐2’‐deoxyuridine (BrdU)	Sigma Aldrich	Cat #B5002‐5G	10 mg/mL in sterile saline
Chemical compound, drug	Lipopolysaccharide (LPS)	Sigma Aldrich	Cat #L4005	Serotype O55:B5 0.5 mg/mL in sterile saline
Chemical compound	Sodium fluorescein (NaF)	Sigma Aldrich	Cat #F6377	0.4 kDa 100 mg/mL in sterile saline
Chemical compound	Paraformaldehyde (32% stock)	Electron Microscopy Sciences	Cat #15714S	4% working solution made in PBS
Chemical compound	Sucrose	Fisher Scientific	Cat #S5‐3	30% w/v working solution made in PBS
Chemical compound	Ethylene glycol	Fisher Scientific	Cat #E178‐4	
Chemical compound	Glycerol	Sigma Aldrich	Cat #G5516	
Chemical compound	Ethylenediaminetetraacetic acid (EDTA)	Boston BioProducts	Cat #BM‐711	
Commercial assay or kit	Pierce BCA Protein Assay Kit	Thermo Scientific	Cat #23227	
Commercial assay or kit	Vectastain ABC Kit	Vector Laboratories	Cat #PK‐4000	
Commercial assay or kit	RNeasy Mini Kit	Qiagen	Cat #74106	
Commercial assay or kit	Superscript III First‐Strand Synthesis SuperMix Kit	Invitrogen	Cat #11752050	
Commercial assay or kit	Applied Biosystems SYBR Green PCR Master Mix	Fisher Scientific	Cat # 43‐091‐55	
Commercial assay or kit	Applied Biosystems TaqMan Multiplex Master Mix	Fisher Scientific	Cat # 44‐842‐63	
Sequence‐based reagent	Mouse *Cd11b* qPCR primers	Integrated DNA Technologies, Inc.		TGGCCTATACAAGCTTGGCTTT/ AAAGGCCGTTACTGAGGTGG
Sequence‐based reagent	Mouse *Clcf1* qPCR primers	Integrated DNA Technologies, Inc.		GACTCGTGGGGGATGTTAGC/ CTAAGCTGCGGAGTTGATGCT
Sequence‐based reagent	Mouse *Dcx* qPCR primers	Integrated DNA Technologies, Inc.		CTTTTGGTTCAGCAGAAGGG/ CAAATGTTCTGGGAGGCACT
Sequence‐based reagent	Mouse *Dlg4* qPCR primers	Integrated DNA Technologies, Inc.		CGCTACCAAGATGAAGACACG/ CAATCACAGGGGGAGAATTG
Sequence‐based reagent	Mouse *Gbp2* qPCR primers	Integrated DNA Technologies, Inc.		TGGGGTAGACGATTCCGCTAA/ AGAAGTGACGGGTTTTCCGTT
Sequence‐based reagent	Mouse *H2d1* qPCR primers	Integrated DNA Technologies, Inc.		TCCGAGATTGTAAAGCGTGAAGA/ ACAGGGCAGTGCAGGGATAG
Sequence‐based reagent	Mouse *Iigp1* qPCR primers	Integrated DNA Technologies, Inc.		GGGGCAATAGCTCATTGGTA/ ACCTCGAAGACATCCCCTTT
Sequence‐based reagent	Mouse *Il1a* qPCR primers	Integrated DNA Technologies, Inc.		TCTCAGATTCACAACTGTTCGTG/ AGAAAATGAGGTCGGTCTCACTA
Sequence‐based reagent	Mouse *Il4* qPCR primers	Integrated DNA Technologies, Inc.		GGTCTCAACCCCCAGCTAGT/ GCCGATGATCTCTCTCAAGTGAT
Sequence‐based reagent	Mouse *Nfkb* qPCR primers	Thermo Fisher	Cat #4331182 Assay ID: Mm00476361_m1	
Sequence‐based reagent	Mouse *S1pr3* qPCR primers	Integrated DNA Technologies, Inc.		AAGCCTAGCGGGAGAGAAAC/ TCAGGGAACAATTGGGAGAG
Sequence‐based reagent	Mouse *Steap4* qPCR primers	Integrated DNA Technologies, Inc.		CCCGAATCGTGTCTTTCCTA/ GGCCTGAGTAATGGTTGCAT
Sequence‐based reagent	Mouse *Syn1* qPCR primers	Integrated DNA Technologies, Inc.		GGAAGGGATCACATTATTGAGG/ TGCTTGTCTTCATCCTGGTG
Sequence‐based reagent	Mouse *Tnfa* qPCR primers	Thermo Fisher	Cat #4331182 Assay ID: Mm00443258_m1	
Sequence‐based reagent	Mouse *Tuj1* qPCR primers	Integrated DNA Technologies, Inc.		TAGACCCCAGCGGCAACTAT/ GTTCCAGGTTCCAAGTCCACC
Software, algorithm	CleverSys	CleverSys, Inc.	RRID: SCR_017141	
Software, algorithm	ANY‐maze	Stoelting Co.	RRID: SCR_014289	
Software, algorithm	Zen	Zeiss	Zen Blue 2.5 RRID: SCR_013672	
Software, algorithm	Image‐Pro	Media Cybernetics, Inc.	Image‐Pro 9.2 RRID: SCR_016879	
Software, algorithm	ImageJ	National Institutes of Health	RRID:SCR_003070	
Software, algorithm	SynapseCounter (ImageJ plugin)		https://github.com/SynPuCo/SynapseCounter	
Software, algorithm	QuantStudio	Applied Biosystems	QuantStudio 6 RRID: SCR_020239	
Software, algorithm	Image Lab	Bio‐Rad	Image Lab 6.0 RRID: SCR_014210	
Software, algorithm	GraphPad Prism	GraphPad Software, Inc.	Graphpad Prism 8 RRID: SCR_002798	

### 2.3 Behavior

#### 2.3.1 Y‐maze cognition

For the spatial recognition task Y‐maze, a Y‐shaped apparatus was constructed with extruded PVC (Komatex). Each arm was 15 in. long and 3 in. wide with 6 in. tall walls. Unique cues in the form of black shapes were adhered to the walls at the ends of two of the arms, while the third arm was un‐cued and designated as the starting point for the mice. Mice were habituated to a dimly lit room for at least 30 min prior to the start of training. First, mice were individually placed in the starting arm and allowed to explore only one of the other two arms (familiar arm) for 5 min; the second arm (novel arm) was blocked off with an acrylic plastic wall identical to that of the rest of the apparatus. After 24 h, each mouse was then returned to the maze with both arms now open to explore for 5 min. All movements were recorded and tracked for analysis using CleverSys Software (CleverSys, Reston, VA). The number of entries into and the time spent in each of the two arms, familiar and novel, was measured. After each trial, the maze was wiped down thoroughly with 70% ethanol. Animals of both ages were run together, and the experimenter was blinded to the age of the animals while performing and analyzing the experiment.

#### 2.3.2 Y‐maze ambulation

To measure distance and velocity, the same Y‐maze protocol was used as described in section 2.3.1. However, all movements were recorded and tracked for analysis using ANY‐maze software (Stoelting Co., Wood Dale, IL), which allows for measurement of the total distance and velocity for the duration of the test. Animals of both ages were run together, and the experimenter was blinded to the age of the animals while performing and analyzing the experiment.

#### 2.3.3 Barnes maze

The Barnes maze is a circular maze with a diameter of 118 cm approximately 95 cm off the ground, consisting of 40 holes with a diameter of 5 cm aligned in three concentric circles. Each day, a hole was designated as the escape hole, where a small black box was placed beneath the hole and provided a space below the maze that the mouse could climb into. To create an aversive environment and motivation to find the escape hole, the maze was illuminated with two large flood lights and a fan blew over the maze, creating palpable wind and a constant background noise of approximately 60 Hz. Two walls and two curtains surrounded the maze, each of which displayed distinct visual cues. Mice were habituated to the room for at least 20–30 min prior to the start of testing. The testing ran for four consecutive days, with five trials each day. Mice were given 90 s to find and enter the escape hole after being placed in the center of the maze. If mice failed to identify the escape hole in that time, they were guided to the hole and encouraged to stay inside for 30 s. The inter‐trial latency was 10 min. For the first 2 days of training (trials 1–10), the escape hole remained unchanged. For the second 2 days of testing (trials 11–20), the escape hole location was changed at the start of each day but was kept consistent for the trials occurring on that day (11‐15, 16–20). Analysis began as soon as the mouse was placed in the center of the maze and concluded either once the mouse was inside the escape hole for >3 s or at a duration of 90 s. After each trial, the maze and escape hole were wiped down thoroughly with 70% ethanol. All movements were recorded and tracked for analysis using CleverSys Software. Animals of both ages were run together, and the experimenter was blinded to the age of the animals while performing and analyzing the experiment. The Barnes maze assay was performed in the same cohort of mice (cohort 2) as the Y‐maze experiment, and these behavioral tests were run approximately 1 week apart.

#### 2.3.4 Grip strength

Mice were habituated to the room for at least 20 min prior to testing. After habituation, each mouse was gently lifted by the base of the tail to the height of the grip bar and allowed to grab the bar with an overhand grip. The mouse was gently pulled to ensure a tight grip and then continuously pulled at a slow, constant horizontal speed until the grip was broken. Steps were repeated for a total of four trials per mouse and peak tension (grams of force) was recorded for each mouse using a grip strength meter (Columbus Instruments, Columbus, OH). At the end of the testing, the body weight of each mouse was recorded. The average pull for each mouse was calculated and normalized to body weight.

### 2.4 Histology

Mice were anesthetized with 2,2,2‐tribromoethanol (Avertin, T48402‐25G, Sigma Aldrich) and subsequently perfused with 0.9% saline transcardially. The brains were dissected and cut sagittally in two even halves. One half was snap frozen in dry ice for protein and RNA analysis, and the other was fixed in 4% PFA (15714S, Electron Microscopy Sciences, Hatfield, PA) in PBS for use in immunohistochemistry. After 2 days of fixation, the hemibrains were transferred to a 30% sucrose (S5‐3, Fisher Scientific, Hampton, NH) in PBS solution and then changed again after 1 day. Hemibrains were sectioned coronally at 30 μm on a microtome at −22°C. Brain slices were collected sequentially into 12 tubes, so that every 12th section of the hippocampus was represented in a given tube. Brain sections were stored in cryoprotectant media composed of 30% ethylene glycol (E178‐4, Fisher Scientific) and 30% glycerol (G5516, Sigma Aldrich) in a sodium phosphate solution at −20°C until needed for staining.

For fluorescent microscopy, blocking was done on free floating sections in the appropriate serum at 10% in PBS‐Triton 0.5% (215680010, ACROS Organics, Fair Lawn, NJ), unless otherwise noted. Primary antibodies were incubated overnight at 4°C, unless otherwise noted. The appropriate fluorescent secondary antibodies (Invitrogen, Carlsbad, CA) were applied the next day at a concentration of 1:300 for 1 h at room temperature followed by Hoechst (H3570, Invitrogen) at a concentration of 1:10,000 for 10 min. Prolong Gold Antifade Mountant (P36934, Invitrogen) was used to coverslip the slides.

Ki67 antibody (ab15580, Abcam, Cambridge, United Kingdom) was used at a concentration of 1:500 with antigen retrieval in 50 mM Na‐citrate (pH 6) for 10 min at 95°C before blocking. BrdU antibody (ab6326, Abcam) was used at a concentration of 1:500 with antigen retrieval in 2N HCL for 30 min at 37°C before blocking. Ki67‐ and BrdU‐positive cells in the blades of the dentate gyrus (DG) were counted live at 20× magnification on a Leica DM5500 B Upright Microscope (Wetzlar, Germany) by a single experimenter blinded to age. Representative images were acquired using an exposure time of 157.68 ms and gain of 2.5 at 20×.

CD3 antibody (555273, BD Biosciences, San Jose, CA) was used at a concentration of 1:100 and stained together with CD45 antibody (702575, Cell Signaling Technology, Danvers, MA) at a concentration of 1:200 to confirm cell type. Together with dyLight 594‐labeled *Lycopersicon esculentum* (Tomato) lectin (DL‐1177, Fisher Scientific) at 1:200, all primary antibodies were incubated overnight at room temperature. Images were acquired using the Hamamatsu Nanozoomer 2.0HT (Hamamatsu City, Japan) at 20×. Quantification in the hippocampus was done by counting CD3‐CD45‐double positive cells found outside of blood vessels (LECTIN‐negative) and within the vessels (LECTIN‐positive) using Image‐Pro 9.2 software (Media Cybernetics, Rockville, MD) by a single experimenter blinded to age. Due to the high background for T cell marker CD3, the immune cell marker CD45 was used to identify immune cells that were then confirmed to be CD3‐positive T cells at a higher magnification. Quantification in the subventricular zone (SVZ) was done by counting all CD3–CD45‐double positive cells regardless of lectin staining using Image‐Pro software by a single experimenter blinded to age.

CD68 antibody (MCA1957, Bio‐Rad, Oxford, United Kingdom) was used at a concentration of 1:1000 and stained together with Iba1 antibody (019‐19741, Wako Chemicals, Richmond, VA), used at 1:2500. CD68/Iba1 images were acquired using the Hamamatsu Nanozoomer 2.0HT at 20×. Quantification was done using percent thresholded area of the entire hippocampus region using ImagePro software by a single experimenter blinded to age.

GFAP antibody (ab53554, Abcam) was used at a concentration of 1:1000. First, images were acquired using a Zeiss LSM800 confocal microscope. The 6 z‐stack (1 μm step size) images in the CA1 region of the hippocampus were acquired at 40×. Maximum intensity projections of each z‐stack were quantified using ImageJ (National Institutes of Health, Bethesda, MD) for percent GFAP thresholded area and total GFAP cell count. Next, images were acquired using the Axio Scan.Z1 (Zeiss, Oberkochen, Germany) at 20×. For GFAP line profile analysis, 6–12 large descending vessels in the hippocampal CA1 area from each mouse (*n* = 9–12 mice) were quantified in Zen Blue 2.5 (Zeiss) by generating a 60 μm linear ROI to measure the fluorescent intensity profiles across each vessel. Data were analyzed by averaging the intensity of the 20 μm segment along the vessel (vascular) and the 20 μm on either side of the vessel (perivascular).

To stain for synapses, sections were blocked in 10% goat serum with PBS and 1% triton for 1 h followed by PSD‐95 antibody (3450S, Cell Signaling Technology) at 1:250 and Synapsin1/2 antibody (106 006, Synaptic Systems, Goettingen, Germany) at 1:750 overnight at 4°C in 3% goat serum in PBS with 0.3% triton. The 10 z‐stack (0.18 μm step size) images in the CA1 region were acquired using a Zeiss LSM800 with Airyscan at 63X, Airyscan processed using Zen Blue 2.5 (Zeiss), and then quantified using the ImageJ macro SynapseCounter (https://github.com/SynPuCo/SynapseCounter) to measure pre‐synaptic Synapsin1/2 puncta, post‐synaptic PSD‐95 puncta, and juxtaposed signal for synapses.

For light microscopy, blocking was done on free floating sections in the appropriate serum at 10% in PBS‐Triton 0.5%. Doublecortin (DCX) antibody (AB2253, Millipore, Burlington, MA) was used at a concentration of 1:2000 and incubated overnight at 4°C. Biotinylated anti‐guinea pig antibody (BA‐7000, Vector Laboratories, Burlingame, CA) was applied the next day at a concentration of 1:300. Staining visualization was achieved by reaction with the Vectastain ABC kit (PK‐4000, Vector Laboratories) and 3,3′‐diaminobenzidine tetrahydrochloride (DAB, D5905, Sigma Aldrich). Dehydration of the mounted slides was achieved using Citrisolv Clearing Agent (22‐143‐975, Decon Labs, King of Prussia, PA) and slides were coverslipped using Cytoseal (8310‐4, Thermo Scientific, Waltham, MA). The number of DCX‐positive cells in the blades of the DG were counted live on a Leica DM5500 B Upright Microscope at 20× magnification by an experimenter blinded to age. Representative images were acquired with the Hamamatsu Nanozoomer 2.0HT at 20×.

### 2.5 Plasma protein quantifications

Blood was collected by cardiac puncture in syringes containing 250 mM EDTA (BM‐711, Boston BioProducts, Ashland, MA). Plasma was isolated by centrifugation at 1000 x *g* for 15 min at 4°C and immediately frozen on dry ice. Mouse plasma was diluted 1:1 in PBS and then shipped on dry ice to Eve Technologies in Calgary, Canada. Single samples were analyzed using a multi‐plex Luminex technology assay for cytokines and chemokines or cell adhesion molecules. Quantitative data was sent in an Excel sheet after completion of the data acquisition and analysis.

### 2.6 qPCR

RNA was isolated from hippocampal brain tissue using the RNeasy Mini Kit (74106, Qiagen, Hilden, Germany) according to the manufacturer's instructions. Briefly, tissue was homogenized in RLT buffer using a Bead Ruptor (Omni International, Kennesaw, GA), and then RNA was bound to an RNA isolation column, washed, and eluted. Contaminating DNA was removed by DNase digestion and cDNA was generated using the Superscript III First‐Strand Synthesis SuperMix Kit (11752050, Invitrogen). A master mix for qPCR was made using SYBR green reagent (43‐091‐55, Fisher Scientific) or TaqMan multiplex reagent (44‐842‐63, Fisher Scientific) and the appropriate forward and reverse primers, and the reactions were run in technical triplicates. The reaction was run on a QuantStudio Flex Real‐Time PCR System (Applied Biosystems, Foster City, CA) and analyzed using the std ddCT protocol on the QuantStudio 6 software (Applied Biosystems) by a single experimenter blinded to age.

### 2.7 Western blot

Cortical lysates were homogenized in RIPA buffer (89901, Thermo Scientific) containing a protease and phosphatase inhibitor cocktail (78446, Thermo Scientific). Tissue was homogenized using the Bead Ruptor, homogenates were centrifuged at max speed (∼21,330 x *g*) for 10 min at 4°C, and then supernatants were collected for subsequent analysis of the soluble fraction. The Pierce BCA protein assay kit (23227, Thermo Scientific) was used to determine protein concentration and lysates were prepared in lithium dodecyl sulfate (LDS) buffer (B0007, Invitrogen). The 25 μg lysate samples were run on Bolt 4–12% Bis‐Tris Plus Gels (NW04125BOX, Invitrogen) and transferred to nitrocellulose membranes using the Trans‐Blot Turbo Mini 0.2 μm nitrocellulose transfer pack (1704158, Bio‐Rad) with the turbo transfer method. Membranes were blocked in 5% milk (1706404, Bio‐Rad) for 1 h at room temperature, then probed with antibodies to Aquaporin‐4 (AQP4, ABN910, Millipore) at 1:500, CD13 (AF2335, R&D Systems, Minneapolis, MN) at 1:500, and Actin‐HRP (13E5 5125, Cell Signaling Technology) at 1:5000 in 5% milk overnight at 4°C. PageRuler Plus Prestained Protein Ladder 10 to 250 kDa (26619, Thermo Scientific) was used as the standard. Blots were imaged following incubation with HRP‐conjugated secondary antibodies at 1:5000 (A16035, A15999, Fischer Scientific) for 1 h at room temperature and subsequently with SuperSignal West Pico PLUS Chemiluminescent Substrate (34580, Thermo Scientific). Blots were imaged on a Bio‐Rad Chemidoc and quantified using Image Lab 6.0 (Bio‐Rad) software. Samples were randomized across gels and run blinded in single replicates. A bridging sample was run to normalize across multiple blots, and band intensities of AQP4 and CD13 were additionally normalized to Actin loading control.

### 2.8 Statistical analysis

All data were analyzed using GraphPad Prism 8 (GraphPad Software, San Diego, CA). Sample sizes were similar to those employed in the field and all experimental *n* values reflect biological replicates of individual mice unless otherwise stated. For *n* > 10 with normally distributed data, parametric tests were used, and for *n* < 10 and data with a non‐normal distribution, non‐parametric tests were used. If technical replicates were used, it is stated explicitly within the methods section. Technical replicates reflect samples replicates from the same mouse, such as ROI. Statistical significance was defined as *p* <0.05.

When two groups were compared in the motor and cognitive tests, data were analyzed using a Mann–Whitney *U* test. Average maximum grip strength across 4 trials was normalized to individual mouse body weight and then analyzed using a mixed‐effects analysis with repeated measures with main effects of age and trial, followed by Mann–Whitney test. For Y‐maze performance, two separate cohorts of mice were run and data were pooled across two experiments. Data were analyzed using a three‐way repeated measures ANOVA for interaction between arm x age x experiment followed by Wilcoxon matched‐paired signed rank tests. For Barnes maze performance, data were tested first for a normalized distribution and then analyzed using a mixed‐effects analysis with repeated measure with main effects of age and trial.

The total number of DCX‐positive cells per DG was estimated by counting the number of positive cells from 6 tissue sections and multiplying the sum of the number counted per section by 12, as an estimate for the total hippocampal volume. Mice with less than 6 quantifiable sections were excluded from the analysis. The thresholded percent area of CD68 and Iba1 were measured from 5–6 hippocampi per mouse using Image‐Pro 9.2 software (Media Cybernetics). Mice with less than 5 quantifiable sections were excluded from the analysis. Ki67‐ and BrdU‐positive cells were counted from 5 dentate gyri per mouse and CD3‐CD45‐double positive cells were counted from the hippocampus and SVZ of 5 hemibrain sections per mouse, and then the counts were summed. Mice with less than 5 quantifiable sections were excluded from the analysis. BrdU and Ki67 data were analyzed using nested *t*‐tests. DCX, CD68, Iba1, SVZ CD3/CD45, and hippocampus parenchyma CD3/CD45 data were analyzed using nested one‐way ANOVAs followed by Tukey's multiple comparisons test. CD3‐CD45‐Lectin triple positive data in the hippocampus was analyzed using Kruskal–Wallis tests followed by Dunn's multiple comparisons tests as data for each individual slice was not recorded during analysis of blood vessels. For GFAP percent area and counts, maximum intensity projections of each CA1 ROI z‐stack were thresholded and quantified using ImageJ. Six sections per mouse were imaged and analyzed using nested *t*‐tests. For GFAP line profile analysis, 6–12 large descending vessels in the hippocampal CA1 area from each mouse were quantified in Zen Blue 2.5 (Zeiss) by generating a 60 μm linear ROI to measure the fluorescence intensity profiles across each vessel by a single experimenter blinded to age. Mice with less than six quantifiable vessels were excluded from the analysis. Data were analyzed by averaging the intensity of the 20 μm segment along the vessel (vascular) and the 20 μm on either side of the vessel (perivascular) followed by nested *t*‐tests. Synapses were analyzed from six ROIs in the CA1 hippocampal region from six mice per age using ordinary one‐way ANOVA, followed by unpaired *t* tests for significance between ages with *n* of 36 ROIs per age.

For gene expression, circulating cytokines and cell adhesion molecules, and extravasated hemibrain sodium fluorescein, data were analyzed using Kruskal–Wallis tests followed by Dunn's multiple comparisons test or Mann–Whitney tests. For gene expression, samples were excluded from final analysis if the standard deviation between triplicates was greater than 1. Normality of western blot data was analyzed using Anderson–Darling test, D'Agostino and Pearson test, Shapiro–Wilk test, and Kolmogorov–Smirnov test. Western blot data with a normal distribution and equal variances were analyzed using an unpaired *t*‐test. Otherwise, they were analyzed using a Mann–Whitney *U* test.

## 3 RESULTS

### 3.1 Impaired cognitive and motor function with age

In humans, aging leads to a progressive decline in cognitive function (Klimova et al., 2017) and, in mice, has been shown to cause impairments in cognitive tasks including the Morris and radial arm water mazes and contextual fear conditioning (Murphy et al., 2006; Villeda et al., 2014; Weber et al., 2015). We found that 20‐ to 22‐month‐old aged mice had impairments in the hippocampal‐dependent spatial learning and memory tasks, Y‐maze (Figure 1A) and Barnes maze (Figure 1B), compared to young 2 to 3‐month‐old mice. However, aging also leads to declines in gait, motor function, and strength in both humans (Williams et al., 2019) and C57BL/6J mouse strains (Murphy et al., 2006; Villeda et al., 2014). We tested locomotor function in young and aged mice and showed that aged mice traveled shorter distances (Figure 1C) and had a 62% reduced velocity (Figure 1D) relative to young mice while exploring the Y‐maze. Next, we assessed forearm grip strength between young and aged mice and identified that aged mice generated significantly less pulling force (Figure 1E). For this task, we used 6.5‐month‐old young mice to ensure there was no difference in body weight between groups (Figure 1F). The impairments in motor function and strength with age confound the interpretation of cognition in both the Y‐maze and Barnes maze and highlight one of the challenges with behavior in aged animals. Therefore, we sought to outline molecular and histological changes that occur at the same time as the impairments in cognition and motor function.

### 3.2 Decreased neurogenesis, proliferation, and synaptic density in the hippocampus with age

New neurons are generated within the SVZ and the subgranular zone of the DG throughout adulthood, and this neurogenesis is greatly decreased with healthy aging and in neurodegenerative disease (Horgusluoglu et al., 2017; Kempermann, 2015; Knoth et al., 2010; Kozareva et al., 2019; Kuhn et al.,^1996^; Kuzumaki et al., 2010; Moreno‐Jimenez et al., 2019) and correlates with cognitive status in humans (Moreno‐Jimenez et al., 2019; Tobin et al., 2019) and mice (Kempermann & Gage, 2002; Kozareva et al., 2019; Raber et al., 2004; Saxe et al., 2006). In the DG, these newborn neurons functionally integrate into neuronal networks and contribute to cognitive processing (Kozareva et al., 2019; Toni & Schinder, 2015). To measure neurogenesis, we examined the newborn neuron marker DCX in the DG using histology and show a dramatic decrease by 6 months of age with little neurogenesis occurring by 18–24 months of age (Figure 2A,B). However, using bulk hippocampal qPCR, *Dcx* gene expression was only modestly reduced (Supplementary Fig. 1A), indicating that histology is a more robust readout for age‐related neurogenesis changes. Additionally, the cell proliferation markers Ki67 (Figure 2C,D) and BrdU (Supplementary Fig. 1B‐C) were also reduced by 97–99% in aged DG relative to young.

Age‐related reductions in synaptic density and expression of genes related to synaptic function occur in both humans and rodents, and these changes correlate with cognitive deficits (Bishop et al., 2010; Blalock et al., 2003; Lee et al., 2000; Xu et al., 2018; Yankner et al., 2008). We found that excitatory synaptic density decreased between 12 and 18 months of age in the Schaffer collateral synapses of the CA1 hippocampal region, which is essential for activity‐dependent synaptic plasticity (Bishop et al., 2010), as measured by juxtaposed pre‐synaptic Synapsin and post‐synaptic PSD‐95 (Figure 2E,F). However, the gene expression of *Syn1* and *Dlg4*, the genes encoding Synapsin‐1 and PSD‐95, respectively, were unchanged by qPCR from bulk hippocampal tissue with age (Supplementary Fig. 1D‐E), while gene expression of neuron‐specific *Tuj1* had a small stepwise reduction with age, which is only significant at 24 months of age (Supplementary Fig. 1F). Taken together, these data suggest that histology may be a better readout for the small synaptic changes that occur with healthy aging in mice, while bulk qPCR may be better suited for detecting larger changes to neuronal morphology or number.

### 3.3 Heightened microgliosis and elevated proinflammatory cytokines with age

Neuroinflammation is a major hallmark of aging and disease (Jansen et al., 2019; Mosher & Wyss‐Coray, 2014) and numerous changes in microglia, which are the resident macrophages of the central nervous system, are impacted by animal age, including proliferation (Long et al., 1998), reactivity (Hefendehl et al., 2014), motility (Damani et al., 2011; Hefendehl et al., 2014), gene expression (Harry, 2013; Hart et al., 2012), and secretion of inflammatory cytokines (Ye & Johnson, 1999; Yu et al., 2002). Using CD68 and Iba1 to mark microglia in the hippocampus, we found a stepwise increase in microgliosis with age (Figure 3A,C). Furthermore, there was increased gene expression of the proinflammatory genes *Tnfa*, *Cd11b*, and *Il1a* analyzed by qPCR from bulk hippocampal tissue (Figure 3D,F). Interestingly, while these genes are predominantly expressed by microglia (Bohlen et al., 2017), they did not show the same stepwise progression as histological evaluation, but rather a sharp increase at 12 or 24 months of age. We also identified a subset of inflammatory genes that are unchanged with age, including *Nfkb* and *Il4* (Supplementary Fig. 2A‐B), suggesting that bulk gene expression may not be a robust readout of age‐related microgliosis.

Circulating factors in the blood can have significant impacts on brain health, including neurogenesis, proliferation, myelination, synaptic plasticity, vascular remodeling, and cognition (Katsimpardi et al., 2014; Ruckh et al., 2012; Villeda et al., 2011, 2014). Additionally, the contributions of inflammaging—the small yet persistently increased levels of proinflammatory signaling with age—are becoming increasingly more appreciated (Goronzy & Weyand, 2019; Lopez‐Otin et al., 2013; Salminen et al., 2012). We examined the plasma levels of two circulating cytokines that are known to mediate microglia activation: IP‐10/CXCL10 (Clarner et al., 2015) and MIG/CXCL9 (Ellis et al., 2010), and we found that levels of IP‐10 and MIG increased with age (Supplementary Fig. 2C,D). Taken together, these results suggest that increased microgliosis and heightened expression of a subset of hippocampal and circulating proinflammatory cytokines occur at the same time as age‐related cognitive and motor decline in mice and could be used as molecular or histological readouts.

### 3.4 Changes to astrocytes and pericytes at the neurovascular unit with age

The NVU plays an essential role in maintaining cerebral blood flow and BBB integrity (Zlokovic, 2008). Astrocytes support brain health by interacting with the NVU and other cell types in the brain parenchyma (Colombo & Farina, 2016; Szu & Binder, 2016) and by providing essential growth factors and metabolites (Eidsvaag et al., 2017; Hoddevik et al., 2017; Seifert et al., 2006; Simard & Nedergaard, 2004; Zeppenfeld et al., 2017). Expression of the astrocyte marker glial fibrillary acidic protein (GFAP) increases with age in humans and mice (Kimbroughet al., 2015; Kovacs et al., 2018; Kress et al., 2014; Stichel & Luebbert, 2007; Wruck & Adjaye, 2020; Zhuang et al., 2019), plays an important role in astrogliosis (Faulkner et al., 2004; Lundkvist et al., 2004; McLean & Lane, 1995; Nawashiro et al., 1998; Pekny & Pekna, 2004; Sofroniew & Vinters, 2010), and its increased expression is correlated with Alzheimer's disease (AD) (Wruck et al., 2016). Additionally, astrocytic endfeet are filled with the aquaporin‐4 (AQP4) water channel that forms an essential part of the BBB, regulates fluid exchange (Haj‐Yasein et al., 2011; Kress et al., 2014; Mestre et al., 2017; Sofroniew & Vinters, 2010; Ueno et al., 2019), and is mislocalized in mouse (Bronzuoli et al., 2019; Kimbrough et al., 2015; Kovacs et al., 2018; Kress et al., 2014; Wilcock et al., 2009; Yang et al., 2011) and human (Iliff et al., 2012; Kress et al., 2014; Simon et al., 2018; Siracusa et al., 2019; Wyss‐Coray et al., 2003; Xiao et al., 2014; Zeppenfeld et al., 2017) aging and disease. To determine if overall astrocyte activation or proliferation is changed with age, we measured percent GFAP area and total GFAP cell count in the CA1 region of the hippocampus (Figure 4A,C). There was a slight elevation in GFAP percent area (Figure 4A,B), but no change in total cell number (Figure 4A,C), indicating an increase in astrocyte activation with age, but not cellular proliferation. Gene expression markers of astrocyte activation in vitro have been extensively characterized (Clarke et al., 2018). However, we found no change in bulk qPCR of pan‐reactive astrocyte genes *S1pr3* or *Steap4*; A1‐type reactive astrocyte genes *Gbp2*, *Iigp1*, or *H2d1;* or the A2‐type reactive astrocyte gene *Clcf1* (Supplementary Fig. 3). Next, to evaluate changes in vascular astrocytes more specifically, we examined GFAP expression along a 60 μm linear ROI across the large descending vessels in the CA1 hippocampus, which have previously been shown to be modulated with age (Bronzuoli et al., 2019; Kress et al., 2014). Indeed, a line graph representation of GFAP along the vessels suggests an increase with age (Figure 4D,G). This age‐related increase in GFAP seemed to be largely in the vascular region (Figure 4E), but there was a trending increase in the surrounding perivascular region as well (Figure 4F). There is also an increase in the astrocytic endfoot protein AQP4 measured from total cortical lysates by western blot (Figure 4H,J).

Pericytes line the capillary walls and interact directly with the endothelial cells of the NVU (Armulik et al., 2005; Diaz‐Flores et al., 2009). In adults, pericytes control capillary diameter (Peppiatt et al., 2006; Yemisci et al., 2009) and BBB integrity (Bell et al., 2010). Furthermore, age‐dependent pericyte loss in animals and humans leads to increased neuroinflammation and leakiness of serum proteins across the BBB (Bell et al., 2010; Rustenhoven et al., 2017). However, high‐quality staining and quantification for pericytes and other makers of the NVU, such as tight junction proteins, often requires cryostat sectioning or transgenic labeled mouse strains (Bell et al., 2010), which is time‐consuming and not available for all labs. Using western blot, we identified a 20% reduction in the brain‐specific pericyte marker CD13 in aged cortical lysates relative to young (Figure 4I,J). Taken together, these changes in astrocytes and pericytes at the NVU may contribute to impaired BBB integrity and identify the molecular or histological tools that can be used to assess these changes.

### 3.5 Increased T cell infiltration into the brain with age

One consequence of inflammaging and BBB dysfunction is the increase in infiltrating T cells into the brain in both humans (Dulken et al., 2019; Gemechu & Bentivoglio, 2012; Loeffler et al., 2011; Moreno‐Jimenez et al., 2019; Moreno‐Valladares et al., 2020) and mice (Dulken et al., 2019; Gemechu & Bentivoglio, 2012; Mrdjen et al., 2018; Ritzel et al., 2016; Stichel & Luebbert, 2007). Susceptibility to T cell infiltration is partially related to the BBB leakiness of the brain region (Loeffler et al., 2011), and infiltration of T cells is greatly enhanced in human patients with AD (Itagaki et al., 1988; Rogers et al., 1988; Togo et al., 2002), in mouse models of AD (Ferretti et al., 2016; Mrdjen et al., 2018), and following injury (Muzio et al., 2010; J. Wang et al., 2015). Infiltration into the hippocampus and SVZ are of particular interest due to their functions as neurogenic niches. T cells have been identified in the SVZ of aged mouse brains with single cell RNA sequencing (Dulken et al., 2019; Ogrodnik et al., 2021) and an increase in cytotoxic CD8+ T cells have been found in various regions of the aged mouse brain by histology (Propson et al., 2021). We used histological markers to quantify T cells in the hippocampus and SVZ across age. There was a stepwise increase in CD3+CD45+ T cells within the hippocampal parenchyma (Figure 5A,D) and within blood vessels (Figure 5B,D) with increasing age. Additionally, there was a large increase in T cells at the SVZ with age (Figure 5C), suggestive of BBB impairment or recruitment of peripheral immune cells to the brain during aging.

### 3.6 High‐dose LPS induces BBB impairment

While BBB impairment in aged humans is well known (Montagne et al., 2015), changes to the BBB in aged mice are less well characterized and the impairment in BBB leakiness is reported to be less robust (Sumbria et al., 2018). To measure BBB leakiness, we administered sodium fluorescein (NaF, 0.4 kDa) by IV tail vein injection and examined fluorescence in brain tissue 4 h later. Indeed, we found that aged mice (22 month) do not have overt BBB leakiness compared to younger (4 month) animals (Figure 6A). To determine if aged mice may be more susceptible to BBB damage, we used a high, acute dose of lipopolysaccharide (LPS, 10 mg/kg), which has previously been reported to increase barrier leakiness 6 h following administration (Bien‐Ly et al., 2015). High‐dose LPS induced leakiness in both young and aged mice, and this leakiness was exacerbated with age (Figure 6A), indicating impaired maintenance of the BBB in aged mice following chemical insult.

LPS has been well studied across multiple labs due to its potent effects and relative ease of use in animal models. LPS administration causes hundreds of genes to be differentially expressed (Chen et al., 2020). Furthermore, LPS increases soluble plasma levels of cell adhesion molecules (CAMs), which are released from endothelial cells in response to damage (Gotsch et al., 1994; Kisucka et al., 2009; Ley et al., 2007; Petri et al., 2008; Rossi et al., 2011). For example, P‐selectin is increased following acute neuroinflammation and blocking it prevents neutrophil recruitment into the brain parenchyma (Bernardes‐Silva et al., 2001) and leads to improved BBB integrity (F. Wu et al., 2015). We identified that high‐dose LPS leads to significant increases in soluble E‐Selectin, ICAM‐1, and P‐Selectin in the plasma of both young and aged mice (Figure 6B,D), suggesting widespread endothelial damage in response to LPS.

## 4 DISCUSSION

We identified changes in neurogenesis, proliferation, synaptic density, microgliosis, neuroinflammation, astrocytes, and pericytes at the NVU, and T cell infiltration into the brain during healthy aging in male C57BL/6J mice and propose the specific techniques that can be used to quantify these changes. Due to the many challenges with cognitive and behavioral testing in mice, we propose these molecular and histological changes may be used as readouts associated with aging‐related cognitive and motor decline. The challenges of measuring behavior in aged mice include optimization of protocols, specialized equipment, and variability within and between aged cohorts. Furthermore, interpreting cognitive decline in aged mice is complicated by the fact that aged animals also have motor impairments. The readouts for many cognitive tasks are influenced by both cognition and ambulation. Finally, blinding of behavioral experiments is confounded by the obvious differences in size and appearance between young and aged animals. Here we aim to form a comprehensive profile of the molecular and histological changes that are robustly modulated with aging in male C57BL/6J mice and more straightforward to implement across labs.

The readouts outlined here support a model of inflammaging and reveal a high level of cross‐talk between modalities. For example, adult neurogenesis is regulated by metabolic factors, the vascular system, and the immune system, which are all modulated with aging (Horgusluoglu et al., 2017; Villeda et al., 2011). Astrocytes express and regulate signaling factors and cytokines (Horgusluoglu et al., 2017; Kozareva et al., 2019; Sofroniew, 2009), while microglia can enhance or suppress neurogenesis under different conditions, contributing to these age‐related changes in neurogenesis (Belarbi & Rosi, 2013; De Lucia et al., 2016; Sierra et al., 2014). Pericytes are lost with aging in rodents and humans leading to increased neuroinflammation (Bell et al., 2010; Rustenhoven et al., 2017) and contributing to dementia (Bowman et al., 2007; Janelidze et al., 2017; Montagne et al., 2015; Sweeney et al., 2018; van de Haar et al., 2016), while improving BBB function is associated with beneficial effects (Dempsey et al., 2000; Kamat et al., 2016; Montagne et al., 2015; Sweeney et al., 2018; Van Skike et al., 2018; Zeppenfeld et al., 2017). Microglia and astrocytes secrete factors that impact BBB permeability and lead to changes in tight junction proteins (Palmer & Ousman, 2018). Overexpression of GFAP in Alzheimer's disease, Parkinson's disease, and healthy patients is correlated with myelin impairment (Han et al., 2019), and astrogliosis can inhibit axonal regeneration (Sofroniew & Vinters, 2010). Additionally, reactive astrogliosis is regulated by several growth factors and cytokines, including TNFα and IL‐1α (Sofroniew, 2009), which we found were also increased in the plasma of mice with age. Finally, T cells may be recruited to the brain by reactive astrocytes (Aloisi et al., 2000) and subsequently release cytokines that trigger microglial activation (Gemechu & Bentivoglio, 2012; Ritzel et al., 2016). Infiltrating T cells may exert effects on cognition through modulation of inflammation (Butovsky et al., 2006; Dulken et al., 2019; Guo et al., 2010; Pluchino et al., 2008; T. Wang et al., 2010; Y. Wang et al., 2008) or neurogenesis (Beers et al., 2011; Gendelman & Appel, 2011; Li et al., 2013; Reynolds et al., 2007; Rezai‐Zadeh et al., 2009). Here, we propose that these changes may be used as molecular and histological endpoints that correspond with aging‐related cognitive and motor decline. Additionally, we identified a number of readouts that were unchanged with age and have limited utility as robust markers of aging in male C57BL/6J mice.

There are a few limitations to the results presented here. We are only reporting results from male mice in the one strain C57BL/6J. As a result, these conclusions can only be generalized within this population of animals. Others have published the differences in female mice or across different aged strains, and we point the reader to these published studies for additional references (Adelof et al., 2019; Kohama et al., 1995; Tran et al., 2021; Weber et al., 2015; Xiong et al., 2018). Broadly, the results reported here across the three modalities of neurons, microglia, and NVU cell types are recapitulated in other strains of mice and across sex. However, the specific timelines and magnitudes are distinct between background strain and sex. While many of these endpoints have been previously reported, the additional data here, and the bringing together of multiple biological mechanisms, is significant as aging is a multimodal process and must be considered holistically. These results, along with reports from the literature, summarized in Table 1, are essential tools for understanding aging processes and development of therapeutics for gerontological disease.

**TABLE 1:** Age‐specific changes in male C57BL/6J mice

Modality	Change with aging	Current study	Citations
Behavior			
*Motor activity*	Reduced speed and distance	Figure 1A‐D	(Boyer, Jaouen, Ibrahim, & Gascon, 2019; Weber et al., 2015; Whitehead et al., 2014)
*Grip strength*	Reduced	Figure 1E,F	(Murphy et al., 2006; Villeda et al., 2014)
*Cognition: MWM, RAWM, CFC, BM, Y‐maze*	Impaired	Figure 1A,B, *	(Murphy et al., 2006; Sukoff Rizzo et al., 2018; Sukoff Rizzo & Silverman, 2016; Villeda et al., 2014; Weber et al., 2015)
Neurons			
*Neurogenesis and proliferation*	Reduced	Figure 2A‐D; Sup Figure 1A‐C	(Horgusluoglu et al., 2017; Kempermann, 2015; Kempermann & Gage, 2002; Kozareva et al., 2019; Villeda et al., 2011)
*Synaptogenesis/synaptic density*	Reduced	Figure 2E,F; Sup Figure 1D,E	(Cizeron et al., 2020; Lee et al., 2000; Morrison & Baxter, 2012; Weber et al., 2015; Xu et al., 2018; Yankner et al., 2008)
*Neurodegeneration*	Unchanged	Not tested	(Kerrisk & Koleske, 2013; Lutz & Osborne, 2013; T. Wu et al., 2019)
Microglia			
*Phagocytosis*	Impaired	Not tested	(Mosher & Wyss‐Coray, 2014)
*Proliferation*	Increased	Figure 3A‐C	(Long et al., 1998; Weber et al., 2015)
*Dystrophy*	Activated shape, increased size	Figure 3A‐C	(Hefendehl et al., 2014)
*Movement*	Decreased	Not tested	(Damani et al., 2011; Hefendehl et al., 2014)
*Signaling*	Altered	Suppl. Figure 2C,D	(Clarner et al., 2015; Ellis et al., 2010; Harry, 2013; Hart et al., 2012; Kawanokuchi et al., 2008; Rock et al., 2005; Shen, Zhang, & Bhat, 2006; Ye & Johnson, 1999; Yu et al., 2002)
*Gene expression*	*Tnfa*, *Cd11b*, *Il1a* increased	Figure 3D‐F	(Schaum et al., 2020; Tabula Muris, 2020)
*Gene expression*	*Nfkb*, *Il4* unchanged	Suppl. Figure 2A,B	
Astrocytes			
*Reactivity*	Increased	Figure 4A‐F	(Boisvert et al., 2018; Clarke et al., 2018; Kohama et al., 1995; Kress et al., 2014; Lynch et al., 2010; O'Callaghan & Miller, 1991; Stichel & Luebbert, 2007; Weber et al., 2015; Zhuang et al., 2019)
*AQP4 Expression*	Mislocalized	Figure 4H,J	(Bronzuoli et al., 2019; Kress et al., 2014)
*Morphology*	Increased size	Figure 4A‐C	(Grosche et al., 2013; Matias, Morgado, & Gomes, 2019; Rodriguez et al., 2014; Verkhratsky, Zorec, Rodriguez‐Arellano, & Parpura, 2019)
*Signaling*	Altered	Not tested	(Boisvert et al., 2018; Clarke et al., 2018; Palmer & Ousman, 2018; Tarantini, Tran, Gordon, Ungvari, & Csiszar, 2017; Verkhratsky et al., 2019)
*Neural modulation*	Synapse elimination	Not tested	(Boisvert et al., 2018; Clarke et al., 2018; Palmer & Ousman, 2018)
*Antigen presentation*	Increased	Not tested	(Orre et al., 2014; Palmer & Ousman, 2018)
*Gene expression*	*S1pr3*, *Steap4*, *Gbp2*, *Iigp1*, *H2d1*, *Clcf1* unchanged	Suppl. Figure 3	(Clarke et al., 2018; Schaum et al., 2020; Tabula Muris, 2020)
Pericytes			
*Cell number and signaling*	Decreased	Figure 4I,J	(Bell et al., 2010; Diaz‐Flores et al., 2009)
T cells			
*CNS infiltration*	Increased	Figure 5	(Dulken et al., 2019; Gemechu & Bentivoglio, 2012; Mrdjen et al., 2018; Ogrodnik et al., 2021; Propson et al., 2021; Ritzel et al., 2016; Stichel & Luebbert, 2007)
*Signaling*	Altered	Not tested	(Desdin‐Mico et al., 2020; Dulken et al., 2019; Ferretti et al., 2016; Ritzel et al., 2016)
Blood‐brain barrier			
*Leakage*	Unchanged	Figure 6A	(Sumbria et al., 2018) (Peppiatt et al., 2006; Rustenhoven et al., 2017)
*Induced leakage*	Increased permeability	Figure 6A	(Bien‐Ly et al., 2015).
*Signaling*	Altered	Figure 6B‐D	(Bernardes‐Silva et al., 2001; Gotsch et al., 1994; Kisucka et al., 2009; Ley et al., 2007; Petri et al., 2008; Rossi et al., 2011; F. Wu et al., 2015)
Brain endothelial cells			
*Signaling and gene expression*	Altered	Figure 6B‐D	(Chen et al., 2020; Marques, Sousa, Sousa, & Palha, 2013; Schaum et al., 2020; Tabula Muris, 2020; Yousef et al., 2019)

Summary of behavioral, molecular, and histological age‐related changes.

* Cognition reported in Figure 1A,B is confounded by motor impairments in aged mice.

## CONFLICT OF INTEREST

All authors were full‐time employees of Alkahest, Inc. at the time they contributed to the experiments in this manuscript.

### PEER REVIEW

The peer review history for this article is available at: https://publons.com/publon/10.1002/brb3.2736.

## Supporting information



Supplementary Fig. 1. Neurogenesis and proliferation are reduced in aged mice while expression of synaptic genes remains unchanged in the hippocampus. (A) Average hippocampal *Dcx* gene expression relative to *Gapdh* measured by SYBR qPCR in mice 3–24 months of age. n = 9‐14 mice per group. Kruskal‐Wallis test p = 0.0045, followed by Dunn's multiple comparisons test: 3 vs. 12 p = 0.2026, 3 vs. 18 p = 0.4264, 3 vs. 24 **p = 0.0028, 12 vs. 18 p>0.9999, 12 vs. 24 p>0.9999, 18 vs. 24 p = 0.3888. (B) Young (2 month) and aged (24 month) mice were dosed with saturating amounts of BrdU IP (2 months ‐ 500mg/kg; 24 months ‐ 150mg/kg) daily for 7 days to label proliferating cells then sacrificed 24 hours following the final injection to assess the number of BrdU‐positive cells per dentate gyrus (DG). n = 5‐12 mice per group. Nested t‐test F = 156.9, ****p<0.0001. (C) Representative images of BrdU staining (green) in the DG (outlined) of young (2 month) and aged (24 month) mice. Scale bar 100mm. (D) Average hippocampal *Syn1* gene expression relative to *Gapdh* measured by SYBR qPCR in mice 3–24 months of age. n = 9‐14 mice per group. Kruskal‐Wallis test p = 0.2266. (E) Average hippocampal *Dlg4* gene expression relative to *Gapdh* measured by SYBR qPCR in mice 3–24 months of age. n = 8‐14 mice per group. Kruskal‐Wallis test p = 0.2879. (F) Average hippocampal *Tuj1* gene expression relative to *Gapdh* measured by SYBR qPCR in mice 3–24 months of age. n = 9‐14 mice per group. Kruskal‐Wallis test p = 0.0161, followed by Dunn's multiple comparisons test: 3 vs. 12 p = 0.4602, 3 vs. 18 p = 0.2752, 3 vs. 24 *p = 0.0118, 12 vs. 18 p>0.9999, 12 vs. 24 p>0.9999, 18 vs. 24 p>0.9999. All data are shown as mean ± s.e.m. Abbreviations: *Dcx*, doublecortin; *Gapdh*, glyceraldehyde‐3‐phosphate dehydrogenase; qPCR, quantitative polymerase chain reaction; BrdU, 5‐bromo‐2’‐deoxyuridine; IP, intraperitoneal; DG, dentate gyrus; *Syn1*, synapsin 1; *Dlg4*, discs large MAGUK scaffold protein 4; *Tuj1*, tubulin beta 3 class III.

Supplementary Fig. 2. Proinflammatory cytokines are elevated with age while RNA expression of some microglial genes from bulk hippocampal tissue are unchanged. (A) Average hippocampal *Nfkb* gene expression relative to *Gapdh* measured by Taqman qPCR in mice 3–24 months of age. n = 9‐14 mice per group. Kruskal‐Wallis test p = 0.2192. (B)Average hippocampal *Il4* gene expression relative to *Gapdh* measured by SYBR qPCR in mice 3–24 months of age. n = 6‐10 mice per group. Kruskal‐Wallis test p = 0.1836. (C) Average circulating plasma levels of IP‐10 (pg/mL) measured by Luminex in mice 3–24 months of age. n = 9‐11 mice per group. Kruskal‐Wallis test p = 0.0006, followed by Dunn's multiple comparisons test: 3 vs. 12 *p = 0.0182, 3 vs. 18 **p = 0.0017, 3 vs. 24 ***p = 0.0028, 12 vs. 18 p>0.9999, 12 vs. 24 p>0.9999, 18 vs. 24 p>0.9999. (D) Average circulating plasma levels of MIG (pg/mL) measured by Luminex in mice 3–24 months of age. n = 9‐11 mice per group. Kruskal‐Wallis test p = 0.0021, followed by Dunn's multiple comparisons test: 3 vs. 12 p = 0.2348, 3 vs. 18 **p = 0.0040, 3 vs. 24 **p = 0.0089, 12 vs. 18 p>0.9999, 12 vs. 24 p>0.9999, 18 vs. 24 p>0.9999. All data are shown as mean ± s.e.m. Abbreviations: RNA, ribonucleic acid; *Nfkb*, Nuclear factor kappa beta subunit; *Gapdh*, glyceraldehyde‐3‐phosphate dehydrogenase; qPCR, quantitative polymerase chain reaction; *Il4*, interleukin 4; IP‐10, interferon gamma‐induced protein 10; MIG, monokine induced by gamma interferon.

Supplementary Fig. 3. Expression of astrocytic reactivity genes from bulk hippocampal tissue are unchanged with age. (A) Average hippocampal *S1pr3* gene expression relative to *Gapdh* measured by SYBR qPCR in mice 3–24 months of age. n = 8‐12 mice per group. Kruskal‐Wallis test p = 0.9098. (B) Average hippocampal *Steap4* gene expression relative to *Gapdh* measured by SYBR qPCR in mice 3–24 months of age. n = 9‐11 mice per group. Kruskal‐Wallis test p = 0.2849. (C) Average hippocampal *Gbp2* gene expression relative to *Gapdh* measured by SYBR qPCR in mice 3–24 months of age. n = 9‐14 mice per group. Kruskal‐Wallis test p = 0.7172. (D) Average hippocampal *Iigp1* gene expression relative to *Gapdh* measured by SYBR qPCR in mice 3–24 months of age. n = 8‐13 mice per group. Kruskal‐Wallis test p = 0.7109. (E) Average hippocampal *H2d1* gene expression relative to *Gapdh* measured by SYBR qPCR in mice 3–24 months of age. n = 9‐14 mice per group. Kruskal‐Wallis test p = 0.3997. (F) Average hippocampal *Clcf1* gene expression relative to *Gapdh* measured by SYBR qPCR in mice 3–24 months of age. n = 8‐14 mice per group. Kruskal‐Wallis test p = 0.5791. All data are shown as mean ± s.e.m. Abbreviations: *S1pr3*, sphingosine‐1‐phosphate receptor 3; *Gapdh*, glyceraldehyde‐3‐phosphate dehydrogenase; qPCR, quantitative polymerase chain reaction; *Steap4*, six transmembrane epithelial antigen of prostate 4; *Gbp2*, guanylate binding protein 2; *Iigp1*, interferon‐gamma‐inducible GTPase Ifgga1 protein; *H2d1*, histocompatibility 2 D region locus 1; *Clcf1*, cardiotrophin Like Cytokine Factor 1.

## Data Availability

The data that support the findings of this study are available from the corresponding author upon reasonable request.

## References

[brb32736-bib-0001] Adelof, J. , Ross, J. M. , Lazic, S. E. , Zetterberg, M. , Wiseman, J. , & Hernebring, M. (2019). Conclusions from a behavioral aging study on male and female F2 hybrid mice on age‐related behavior, buoyancy in water‐based tests, and an ethical method to assess lifespan. Aging (Albany NY), 11(17), 7150–7168. 10.18632/aging.102242 31509518 PMC6756906

[brb32736-bib-0002] Aloisi, F. , Ria, F. , & Adorini, L. (2000). Regulation of T‐cell responses by CNS antigen‐presenting cells: Different roles for microglia and astrocytes. Immunol Today, 21(3), 141–147. 10.1016/s0167-5699(99)01512-1 10689302

[brb32736-bib-0003] Armulik, A. , Abramsson, A. , & Betsholtz, C. (2005). Endothelial/pericyte interactions. Circ Res, 97(6), 512–523. 10.1161/01.RES.0000182903.16652.d7 16166562

[brb32736-bib-0004] Bakula, D. , Ablasser, A. , Aguzzi, A. , Antebi, A. , Barzilai, N. , Bittner, M. I. , Jensen, M. B. , Calkhoven, C. F. , Chen, D. , de Grey, A. D. N. J. , Feige, J. N. , Georgievskaya, A. , Gladyshev, V. N. , Golato, T. , Gudkov, A. V. , Hoppe, T. , Kaeberlein, M. , Katajisto, P. , Kennedy, B. K. , … Scheibye‐Knudsen, M. (2019). Latest advances in aging research and drug discovery. Aging (Albany NY), 11(22), 9971–9981. doi:10.18632/aging.102487 31770722 PMC6914421

[brb32736-bib-0005] Beers, D. R. , Henkel, J. S. , Zhao, W. , Wang, J. , Huang, A. , Wen, S. , Liao, B. , & Appel, S. H. (2011). Endogenous regulatory T lymphocytes ameliorate amyotrophic lateral sclerosis in mice and correlate with disease progression in patients with amyotrophic lateral sclerosis. Brain, 134(Pt 5), 1293–1314. 10.1093/brain/awr074 21596768 PMC3097891

[brb32736-bib-0006] Belarbi, K. , & Rosi, S. (2013). Modulation of adult‐born neurons in the inflamed hippocampus. Front Cell Neurosci, 7, 145. 10.3389/fncel.2013.00145 24046730 PMC3764370

[brb32736-bib-0007] Bell, R. D. , Winkler, E. A. , Sagare, A. P. , Singh, I. , LaRue, B. , Deane, R. , & Zlokovic, B. V. (2010). Pericytes control key neurovascular functions and neuronal phenotype in the adult brain and during brain aging. Neuron, 68(3), 409–427. 10.1016/j.neuron.2010.09.043 21040844 PMC3056408

[brb32736-bib-0008] Benveniste, H. , Liu, X. , Koundal, S. , Sanggaard, S. , Lee, H. , & Wardlaw, J. (2018). The Glymphatic System and Waste Clearance with Brain Aging: A Review. Gerontology, 1–14. 10.1159/000490349 PMC6329683 29996134

[brb32736-bib-0009] Bernardes‐Silva, M. , Anthony, D. C. , Issekutz, A. C. , & Perry, V. H. (2001). Recruitment of neutrophils across the blood‐brain barrier: The role of E‐ and P‐selectins. J Cereb Blood Flow Metab, 21(9), 1115–1124. 10.1097/00004647-200109000-00009 11524616

[brb32736-bib-0010] Bettio, L. E. B. , Rajendran, L. , & Gil‐Mohapel, J. (2017). The effects of aging in the hippocampus and cognitive decline. Neurosci Biobehav Rev, 79, 66–86. 10.1016/j.neubiorev.2017.04.030 28476525

[brb32736-bib-0011] Bien‐Ly, N. , Boswell, C. A. , Jeet, S. , Beach, T. G. , Hoyte, K. , Luk, W. , Shihadeh, V. , Ulufatu, S. , Foreman, O. , Lu, Y. , DeVoss, J. , van der Brug, M. , & Watts, R. J. (2015). Lack of widespread BBB disruption in Alzheimer's disease models: Focus on therapeutic antibodies. Neuron, 88(2), 289–297. 10.1016/j.neuron.2015.09.036 26494278

[brb32736-bib-0012] Bishop, N. A. , Lu, T. , & Yankner, B. A. (2010). Neural mechanisms of ageing and cognitive decline. Nature, 464(7288), 529–535. 10.1038/nature08983 20336135 PMC2927852

[brb32736-bib-0013] Blalock, E. M. , Chen, K. C. , Sharrow, K. , Herman, J. P. , Porter, N. M. , Foster, T. C. , & Landfield, P. W. (2003). Gene microarrays in hippocampal aging: Statistical profiling identifies novel processes correlated with cognitive impairment. J Neurosci, 23(9), 3807–3819. 12736351 10.1523/JNEUROSCI.23-09-03807.2003 PMC6742177

[brb32736-bib-0014] Bohlen, C. J. , Bennett, F. C. , Tucker, A. F. , Collins, H. Y. , Mulinyawe, S. B. , & Barres, B. A. (2017). Diverse requirements for microglial survival, specification, and function revealed by defined‐medium cultures. Neuron, 94(4), 759–773. e758. 10.1016/j.neuron.2017.04.043 28521131 PMC5523817

[brb32736-bib-0015] Boisvert, M. M. , Erikson, G. A. , Shokhirev, M. N. , & Allen, N. J. (2018). The aging astrocyte transcriptome from multiple regions of the mouse brain. Cell Rep, 22(1), 269–285. 10.1016/j.celrep.2017.12.039 29298427 PMC5783200

[brb32736-bib-0016] Bowman, G. L. , Kaye, J. A. , Moore, M. , Waichunas, D. , Carlson, N. E. , & Quinn, J. F. (2007). Blood‐brain barrier impairment in Alzheimer disease: Stability and functional significance. Neurology, 68(21), 1809–1814. 10.1212/01.wnl.0000262031.18018.1a 17515542 PMC2668699

[brb32736-bib-0017] Boyer, F. , Jaouen, F. , Ibrahim, E. C. , & Gascon, E. (2019). Deficits in social behavior precede cognitive decline in middle‐aged mice. Front Behav Neurosci, 13, 55. 10.3389/fnbeh.2019.00055 30971905 PMC6445840

[brb32736-bib-0018] Bronzuoli, M. R. , Facchinetti, R. , Valenza, M. , Cassano, T. , Steardo, L. , & Scuderi, C. (2019). Astrocyte function is affected by aging and not Alzheimer's disease: A preliminary investigation in hippocampi of 3xTg‐AD mice. Front Pharmacol, 10, 644. 10.3389/fphar.2019.00644 31244658 PMC6562169

[brb32736-bib-0019] Butovsky, O. , Ziv, Y. , Schwartz, A. , Landa, G. , Talpalar, A. E. , Pluchino, S. , Martino, G. , & Schwartz, M. (2006). Microglia activated by IL‐4 or IFN‐gamma differentially induce neurogenesis and oligodendrogenesis from adult stem/progenitor cells. Mol Cell Neurosci, 31(1), 149–160. 10.1016/j.mcn.2005.10.006 16297637

[brb32736-bib-0020] Chen, M. B. , Yang, A. C. , Yousef, H. , Lee, D. , Chen, W. , Schaum, N. , Lehallier, B. , Quake, S. R. , & Wyss‐Coray, T. (2020). Brain endothelial cells are exquisite sensors of age‐related circulatory cues. Cell Rep, 30(13), 4418–4432. e4414. 10.1016/j.celrep.2020.03.012 32234477 PMC7292569

[brb32736-bib-0021] Cizeron, M. , Qiu, Z. , Koniaris, B. , Gokhale, R. , Komiyama, N. H. , Fransen, E. , & Grant, S. G. N. (2020). A brainwide atlas of synapses across the mouse life span. Science, 369(6501), 270–275. 10.1126/science.aba3163 32527927 PMC7115813

[brb32736-bib-0022] Clarke, L. E. , Liddelow, S. A. , Chakraborty, C. , Munch, A. E. , Heiman, M. , & Barres, B. A. (2018). Normal aging induces A1‐like astrocyte reactivity. Proc Natl Acad Sci U S A, 115(8), E1896–E1905. 10.1073/pnas.1800165115 29437957 PMC5828643

[brb32736-bib-0023] Clarner, T. , Janssen, K. , Nellessen, L. , Stangel, M. , Skripuletz, T. , Krauspe, B. , Hess, F. ‐ M. , Denecke, B. , Beutner, C. , Linnartz‐Gerlach, B. , Neumann, H. , Vallières, L. , Amor, S. , Ohl, K. , Tenbrock, K. , Beyer, C. , & Kipp, M. (2015). CXCL10 triggers early microglial activation in the cuprizone model. J Immunol, 194(7), 3400–3413. 10.4049/jimmunol.1401459 25725102

[brb32736-bib-0024] Colombo, E. , & Farina, C. (2016). Astrocytes: Key regulators of neuroinflammation. Trends Immunol, 37(9), 608–620. 10.1016/j.it.2016.06.006 27443914

[brb32736-bib-0025] Damani, M. R. , Zhao, L. , Fontainhas, A. M. , Amaral, J. , Fariss, R. N. , & Wong, W. T. (2011). Age‐related alterations in the dynamic behavior of microglia. Aging Cell, 10(2), 263–276. 10.1111/j.1474-9726.2010.00660.x 21108733 PMC3056927

[brb32736-bib-0026] De Lucia, C. , Rinchon, A. , Olmos‐Alonso, A. , Riecken, K. , Fehse, B. , Boche, D. , Hugh Perry, V. , & Gomez‐Nicola, D. (2016). Microglia regulate hippocampal neurogenesis during chronic neurodegeneration. Brain Behav Immun, 55, 179–190. 10.1016/j.bbi.2015.11.001 26541819 PMC4907582

[brb32736-bib-0027] Dempsey, R. J. , Baskaya, M. K. , & Dogan, A. (2000). Attenuation of brain edema, blood‐brain barrier breakdown, and injury volume by ifenprodil, a polyamine‐site *N*‐methyl‐D‐aspartate receptor antagonist, after experimental traumatic brain injury in rats. Neurosurgery, 47(2), 399–404. discussion 404‐396. 10.1097/00006123-200008000-00024 10942013

[brb32736-bib-0028] Desdin‐Mico, G. , Soto‐Heredero, G. , Aranda, J. F. , Oller, J. , Carrasco, E. , Gabande‐Rodriguez, E. , Blanco, E. M. , Alfranca, A. , Cussó, L. , Desco, M. , Ibañez, B. , Gortazar, A. R. , Fernández‐Marcos, P. , Navarro, M. N. , Hernaez, B. , Alcamí, A. , Baixauli, F. , & Mittelbrunn, M. (2020). T cells with dysfunctional mitochondria induce multimorbidity and premature senescence. Science, 10.1126/science.aax0860 PMC7616968 32439659

[brb32736-bib-0029] Diaz‐Flores, L. , Gutierrez, R. , Madrid, J. F. , Varela, H. , Valladares, F. , Acosta, E. , Martín‐Vasallo, P. , & Diaz‐Flores, L., Jr (2009). Pericytes. Morphofunction, interactions and pathology in a quiescent and activated mesenchymal cell niche. Histol Histopathol, 24(7), 909–969. doi:10.14670/HH-24.909 19475537

[brb32736-bib-0030] Dulken, B. W. , Buckley, M. T. , Navarro Negredo, P. , Saligrama, N. , Cayrol, R. , Leeman, D. S. , George, B. M. , Boutet, S. P. C. , Hebestreit, K. , Pluvinage, J. V. , Wyss‐Coray, T. , Weissman, I. L. , Vogel, H. , Davis, M. M. , & Brunet, A. (2019). Single‐cell analysis reveals T cell infiltration in old neurogenic niches. Nature, 571(7764), 205–210. 10.1038/s41586-019-1362-5 31270459 PMC7111535

[brb32736-bib-0031] Eidsvaag, V. A. , Enger, R. , Hansson, H. A. , Eide, P. K. , & Nagelhus, E. A. (2017). Human and mouse cortical astrocytes differ in aquaporin‐4 polarization toward microvessels. Glia, 65(6), 964–973. 10.1002/glia.23138 28317216 PMC5413834

[brb32736-bib-0032] Ellis, S. L. , Gysbers, V. , Manders, P. M. , Li, W. , Hofer, M. J. , Muller, M. , & Campbell, I. L. (2010). The cell‐specific induction of CXC chemokine ligand 9 mediated by IFN‐gamma in microglia of the central nervous system is determined by the myeloid transcription factor PU.1. J Immunol, 185(3), 1864–1877. 10.4049/jimmunol.1000900 20585034 PMC2925661

[brb32736-bib-0033] Faulkner, J. R. , Herrmann, J. E. , Woo, M. J. , Tansey, K. E. , Doan, N. B. , & Sofroniew, M. V. (2004). Reactive astrocytes protect tissue and preserve function after spinal cord injury. J Neurosci, 24(9), 2143–2155. 10.1523/JNEUROSCI.3547-03.2004 14999065 PMC6730429

[brb32736-bib-0034] Ferretti, M. T. , Merlini, M. , Spani, C. , Gericke, C. , Schweizer, N. , Enzmann, G. , Engelhardt, B. , Kulic, L. , Suter, T. , & Nitsch, R. M. (2016). T‐cell brain infiltration and immature antigen‐presenting cells in transgenic models of Alzheimer's disease‐like cerebral amyloidosis. Brain Behav Immun, 54, 211–225. 10.1016/j.bbi.2016.02.009 26872418

[brb32736-bib-0035] Gemechu, J. M. , & Bentivoglio, M. (2012). T Cell recruitment in the brain during normal aging. Front Cell Neurosci, 6, 38. 10.3389/fncel.2012.00038 23049498 PMC3446775

[brb32736-bib-0036] Gendelman, H. E. , & Appel, S. H. (2011). Neuroprotective activities of regulatory T cells. Trends Mol Med, 17(12), 687–688. 10.1016/j.molmed.2011.08.005 21996344 PMC5892451

[brb32736-bib-0037] Goronzy, J. J. , & Weyand, C. M. (2019). Mechanisms underlying T cell ageing. Nat Rev Immunol, 19(9), 573–583. 10.1038/s41577-019-0180-1 31186548 PMC7584388

[brb32736-bib-0038] Gotsch, U. , Jager, U. , Dominis, M. , & Vestweber, D. (1994). Expression of P‐selectin on endothelial cells is upregulated by LPS and TNF‐alpha in vivo. Cell Adhes Commun, 2(1), 7–14. 10.3109/15419069409014198 7526954

[brb32736-bib-0039] Grosche, A. , Grosche, J. , Tackenberg, M. , Scheller, D. , Gerstner, G. , Gumprecht, A. , Pannicke, T. , Hirrlinger, P. G. , Wilhelmsson, U. , Hüttmann, K. , Härtig, W. , Steinhäuser, C. , Pekny, M. , & Reichenbach, A. (2013). Versatile and simple approach to determine astrocyte territories in mouse neocortex and hippocampus. PLoS One, 8(7), e69143. 10.1371/journal.pone.0069143 23935940 PMC3720564

[brb32736-bib-0040] Guo, J. , Li, H. , Yu, C. , Liu, F. , Meng, Y. , Gong, W. , Yang, H. , Shen, X. , Ju, G. , Li, Z. , & Wang, J. (2010). Decreased neural stem/progenitor cell proliferation in mice with chronic/nonremitting experimental autoimmune encephalomyelitis. Neurosignals, 18(1), 1–8. 10.1159/000242424 19786810

[brb32736-bib-0041] Haj‐Yasein, N. N. , Vindedal, G. F. , Eilert‐Olsen, M. , Gundersen, G. A. , Skare, Ø. , Laake, P. , Klungland, A. , Thorén, A. E. , Burkhardt, J. M. , Ottersen, O. P. , & Nagelhus, E. A. (2011). Glial‐conditional deletion of aquaporin‐4 (Aqp4) reduces blood‐brain water uptake and confers barrier function on perivascular astrocyte endfeet. Proc Natl Acad Sci U S A, 108(43), 17815–17820. 10.1073/pnas.1110655108 21990350 PMC3203818

[brb32736-bib-0042] Han, F. , Perrin, R. J. , Wang, Q. , Wang, Y. , Perlmutter, J. S. , Morris, J. C. , Benzinger, T. L. S. , & Xu, J. (2019). Neuroinflammation and myelin status in Alzheimer's disease, Parkinson's disease, and normal aging brains: A small sample study. Parkinsons Dis, 2019, 7975407. 10.1155/2019/7975407 31354934 PMC6637678

[brb32736-bib-0043] Harry, G. J. (2013). Microglia during development and aging. Pharmacol Ther, 139(3), 313–326. 10.1016/j.pharmthera.2013.04.013 23644076 PMC3737416

[brb32736-bib-0044] Hart, A. D. , Wyttenbach, A. , Perry, V. H. , & Teeling, J. L. (2012). Age related changes in microglial phenotype vary between CNS regions: grey versus white matter differences. Brain Behav Immun, 26(5), 754–765. 10.1016/j.bbi.2011.11.006 22155499 PMC3381227

[brb32736-bib-0045] Hefendehl, J. K. , Neher, J. J. , Suhs, R. B. , Kohsaka, S. , Skodras, A. , & Jucker, M. (2014). Homeostatic and injury‐induced microglia behavior in the aging brain. Aging Cell, 13(1), 60–69. 10.1111/acel.12149 23953759 PMC4326865

[brb32736-bib-0046] Heinze‐Milne, S. D. , Banga, S. , & Howlett, S. E. (2019). Frailty assessment in animal models. Gerontology, 65(6), 610–619. 10.1159/000501333 31330523

[brb32736-bib-0047] Hoddevik, E. H. , Khan, F. H. , Rahmani, S. , Ottersen, O. P. , Boldt, H. B. , & Amiry‐Moghaddam, M. (2017). Factors determining the density of AQP4 water channel molecules at the brain‐blood interface. Brain Struct Funct, 222(4), 1753–1766. 10.1007/s00429-016-1305-y 27629271 PMC5406442

[brb32736-bib-0048] Hodgson, R. , Kennedy, B. K. , Masliah, E. , Scearce‐Levie, K. , Tate, B. , Venkateswaran, A. , & Braithwaite, S. P. (2020). Aging: Therapeutics for a healthy future. Neurosci Biobehav Rev, 108, 453–458. 10.1016/j.neubiorev.2019.11.021 31783058 PMC9046979

[brb32736-bib-0049] Horgusluoglu, E. , Nudelman, K. , Nho, K. , & Saykin, A. J. (2017). Adult neurogenesis and neurodegenerative diseases: A systems biology perspective. Am J Med Genet B Neuropsychiatr Genet, 174(1), 93–112. 10.1002/ajmg.b.32429 26879907 PMC4987273

[brb32736-bib-0050] Hou, Y. , Dan, X. , Babbar, M. , Wei, Y. , Hasselbalch, S. G. , Croteau, D. L. , & Bohr, V. A. (2019). Ageing as a risk factor for neurodegenerative disease. Nat Rev Neurol, 15(10), 565–581. 10.1038/s41582-019-0244-7 31501588

[brb32736-bib-0051] Iliff, J. J. , Wang, M. , Liao, Y. , Plogg, B. A. , Peng, W. , Gundersen, G. A. , Benveniste, H. , Vates, G. E. , Deane, R. , Goldman, S. A. , Nagelhus, E. A. , & Nedergaard, M. (2012). A paravascular pathway facilitates CSF flow through the brain parenchyma and the clearance of interstitial solutes, including amyloid beta. Sci Transl Med, 4(147), 147ra111. 10.1126/scitranslmed.3003748 PMC3551275 22896675

[brb32736-bib-0052] Itagaki, S. , McGeer, P. L. , & Akiyama, H. (1988). Presence of T‐cytotoxic suppressor and leucocyte common antigen positive cells in Alzheimer's disease brain tissue. Neurosci Lett, 91(3), 259–264. 10.1016/0304-3940(88)90690-8 2972943

[brb32736-bib-0053] Janelidze, S. , Hertze, J. , Nagga, K. , Nilsson, K. , Nilsson, C. , Nilsson, C. , Wennström, M. , van Westen, D. , Blennow, K. , Zetterberg, H. , & Hansson, O. (2017). Increased blood‐brain barrier permeability is associated with dementia and diabetes but not amyloid pathology or APOE genotype. Neurobiol Aging, 51, 104–112. 10.1016/j.neurobiolaging.2016.11.017 28061383 PMC5754327

[brb32736-bib-0054] Jansen, I. E. , Savage, J. E. , Watanabe, K. , Bryois, J. , Williams, D. M. , Steinberg, S. , Sealock, J. , Karlsson, I. K. , Hägg, S. , Athanasiu, L. , Voyle, N. , Proitsi, P. , Witoelar, A. , Stringer, S. , Aarsland, D. , Almdahl, I S. , Andersen, F. , Bergh, S. , Bettella, F. , … Posthuma, D. (2019). Genome‐wide meta‐analysis identifies new loci and functional pathways influencing Alzheimer's disease risk. Nat Genet, 51(3), 404–413. 10.1038/s41588-018-0311-9 30617256 PMC6836675

[brb32736-bib-0055] Kamat, P. K. , Kyles, P. , Kalani, A. , & Tyagi, N. (2016). Hydrogen sulfide ameliorates homocysteine‐induced alzheimer's disease‐like pathology, blood‐brain barrier disruption, and synaptic disorder. Mol Neurobiol, 53(4), 2451–2467. 10.1007/s12035-015-9212-4 26019015 PMC4662933

[brb32736-bib-0056] Kane, A. E. , Keller, K. M. , Heinze‐Milne, S. , Grandy, S. A. , & Howlett, S. E. (2019). A murine frailty index based on clinical and laboratory measurements: Links between frailty and pro‐inflammatory cytokines differ in a sex‐specific manner. J Gerontol A Biol Sci Med Sci, 74(3), 275–282. 10.1093/gerona/gly117 29788087 PMC6376103

[brb32736-bib-0057] Katsimpardi, L. , Litterman, N. K. , Schein, P. A. , Miller, C. M. , Loffredo, F. S. , Wojtkiewicz, G. R. , Chen, J. W. , Lee, R. T. , Wagers, A. J. , & Rubin, L. L. (2014). Vascular and neurogenic rejuvenation of the aging mouse brain by young systemic factors. Science, 344(6184), 630–634. 10.1126/science.1251141 24797482 PMC4123747

[brb32736-bib-0058] Kawanokuchi, J. , Shimizu, K. , Nitta, A. , Yamada, K. , Mizuno, T. , Takeuchi, H. , & Suzumura, A. (2008). Production and functions of IL‐17 in microglia. J Neuroimmunol, 194(1‐2), 54–61. 10.1016/j.jneuroim.2007.11.006 18164424

[brb32736-bib-0059] Kempermann, G. (2015). Activity dependency and aging in the regulation of adult neurogenesis. Cold Spring Harb Perspect Biol, 7(11). 10.1101/cshperspect.a018929 PMC4632662 26525149

[brb32736-bib-0060] Kempermann, G. , & Gage, F. H. (2002). Genetic determinants of adult hippocampal neurogenesis correlate with acquisition, but not probe trial performance, in the water maze task. Eur J Neurosci, 16(1), 129–136. 10.1046/j.1460-9568.2002.02042.x 12153537

[brb32736-bib-0061] Kerrisk, M. E. , & Koleske, A. J. (2013). Arg kinase signaling in dendrite and synapse stabilization pathways: memory, cocaine sensitivity, and stress. Int J Biochem Cell Biol, 45(11), 2496–2500. 10.1016/j.biocel.2013.07.018 23916785 PMC3797846

[brb32736-bib-0062] Kimbrough, I. F. , Robel, S. , Roberson, E. D. , & Sontheimer, H. (2015). Vascular amyloidosis impairs the gliovascular unit in a mouse model of Alzheimer's disease. Brain, 138(Pt 12), 3716–3733. 10.1093/brain/awv327 26598495 PMC5006220

[brb32736-bib-0063] Kisucka, J. , Chauhan, A. K. , Zhao, B. Q. , Patten, I. S. , Yesilaltay, A. , Krieger, M. , & Wagner, D. D. (2009). Elevated levels of soluble P‐selectin in mice alter blood‐brain barrier function, exacerbate stroke, and promote atherosclerosis. Blood, 113(23), 6015–6022. 10.1182/blood-2008-10-186650 19349621 PMC2700332

[brb32736-bib-0064] Klimova, B. , Valis, M. , & Kuca, K. (2017). Cognitive decline in normal aging and its prevention: a review on non‐pharmacological lifestyle strategies. Clin Interv Aging, 12, 903–910. 10.2147/CIA.S132963 28579767 PMC5448694

[brb32736-bib-0065] Knoth, R. , Singec, I. , Ditter, M. , Pantazis, G. , Capetian, P. , Meyer, R. P. , Horvat, V. , Volk, B. , & Kempermann, G. (2010). Murine features of neurogenesis in the human hippocampus across the lifespan from 0 to 100 years. PLoS One, 5(1), e8809. 10.1371/journal.pone.0008809 20126454 PMC2813284

[brb32736-bib-0066] Kobrina, A. , Schrode, K. M. , Screven, L. A. , Javaid, H. , Weinberg, M. M. , Brown, G. , Board, R. , Villavisanis, D. F. , Dent, M. L. , & Lauer, A. M. (2020). Linking anatomical and physiological markers of auditory system degeneration with behavioral hearing assessments in a mouse (Mus musculus) model of age‐related hearing loss. Neurobiol Aging, 96, 87–103. 10.1016/j.neurobiolaging.2020.08.012 32950782 PMC8080312

[brb32736-bib-0067] Kohama, S. G. , Goss, J. R. , Finch, C. E. , & McNeill, T. H. (1995). Increases of glial fibrillary acidic protein in the aging female mouse brain. Neurobiol Aging, 16(1), 59–67. 10.1016/0197-4580(95)80008-f 7723937

[brb32736-bib-0068] Kovacs, G. G. , Yousef, A. , Kaindl, S. , Lee, V. M. , & Trojanowski, J. Q. (2018). Connexin‐43 and aquaporin‐4 are markers of ageing‐related tau astrogliopathy (ARTAG)‐related astroglial response. Neuropathol Appl Neurobiol, 44(5), 491–505. 10.1111/nan.12427 28755467 PMC5788733

[brb32736-bib-0069] Kozareva, D. A. , Cryan, J. F. , & Nolan, Y. M. (2019). Born this way: Hippocampal neurogenesis across the lifespan. Aging Cell, 18(5), e13007. 10.1111/acel.13007 31298475 PMC6718573

[brb32736-bib-0070] Kress, B. T. , Iliff, J. J. , Xia, M. , Wang, M. , Wei, H. S. , Zeppenfeld, D. , Xie, L. , Kang, H. , Xu, Q. , Liew, J. A. , Plog, B. A. , Ding, F. , Deane, R. , & Nedergaard, M. (2014). Impairment of paravascular clearance pathways in the aging brain. Ann Neurol, 76(6), 845–861. 10.1002/ana.24271 25204284 PMC4245362

[brb32736-bib-0071] Kuhn, H. G. , Dickinson‐Anson, H. , & Gage, F. H. (1996). Neurogenesis in the dentate gyrus of the adult rat: Age‐related decrease of neuronal progenitor proliferation. J Neurosci, 16(6), 2027–2033. 8604047 10.1523/JNEUROSCI.16-06-02027.1996 PMC6578509

[brb32736-bib-0072] Kuzumaki, N. , Ikegami, D. , Tamura, R. , Sasaki, T. , Niikura, K. , Narita, M. , Miyashita, K. , Imai, S. , Takeshima, H. , Ando, T. , Igarashi, K. , Kanno, J. , Ushijima, T. , Suzuki, T. , & Narita, M. (2010). Hippocampal epigenetic modification at the doublecortin gene is involved in the impairment of neurogenesis with aging. Synapse, 64(8), 611–616. 10.1002/syn.20768 20340168

[brb32736-bib-0073] Lee, C. K. , Weindruch, R. , & Prolla, T. A. (2000). Gene‐expression profile of the ageing brain in mice. Nat Genet, 25(3), 294–297. 10.1038/77046 10888876

[brb32736-bib-0074] Ley, K. , Laudanna, C. , Cybulsky, M. I. , & Nourshargh, S. (2007). Getting to the site of inflammation: The leukocyte adhesion cascade updated. Nat Rev Immunol, 7(9), 678–689. 10.1038/nri2156 17717539

[brb32736-bib-0075] Li, P. , Gan, Y. , Sun, B. L. , Zhang, F. , Lu, B. , Gao, Y. , Liang, W. , Thomson, A. W. , Chen, J. , & Hu, X. (2013). Adoptive regulatory T‐cell therapy protects against cerebral ischemia. Ann Neurol, 74(3), 458–471. 10.1002/ana.23815 23674483 PMC3748165

[brb32736-bib-0076] Loeffler, C. , Dietz, K. , Schleich, A. , Schlaszus, H. , Stoll, M. , Meyermann, R. , & Mittelbronn, M. (2011). Immune surveillance of the normal human CNS takes place in dependence of the locoregional blood‐brain barrier configuration and is mainly performed by CD3(+)/CD8(+) lymphocytes. Neuropathology, 31(3), 230–238. 10.1111/j.1440-1789.2010.01167.x 21092063

[brb32736-bib-0077] Long, J. M. , Kalehua, A. N. , Muth, N. J. , Calhoun, M. E. , Jucker, M. , Hengemihle, J. M. , Ingram, D. K. , & Mouton, P. R. (1998). Stereological analysis of astrocyte and microglia in aging mouse hippocampus. Neurobiol Aging, 19(5), 497–503. 10.1016/s0197-4580(98)00088-8 9880052

[brb32736-bib-0078] Lopez‐Otin, C. , Blasco, M. A. , Partridge, L. , Serrano, M. , & Kroemer, G. (2013). The hallmarks of aging. Cell, 153(6), 1194–1217. 10.1016/j.cell.2013.05.039 23746838 PMC3836174

[brb32736-bib-0079] Lundkvist, A. , Reichenbach, A. , Betsholtz, C. , Carmeliet, P. , Wolburg, H. , & Pekny, M. (2004). Under stress, the absence of intermediate filaments from Muller cells in the retina has structural and functional consequences. J Cell Sci, 117(Pt 16), 3481–3488. 10.1242/jcs.01221 15226376

[brb32736-bib-0080] Lutz, C. M. , & Osborne, M. A. (2013). Optimizing mouse models of neurodegenerative disorders: are therapeutics in sight? Future Neurol, 9(1), 67–75. 10.2217/fnl.13.66 29479291 PMC5822737

[brb32736-bib-0081] Lynch, A. M. , Murphy, K. J. , Deighan, B. F. , O'Reilly, J. A. , Gun'ko, Y. K. , Cowley, T. R. , Gonzalez‐Reyes, R. E. , & Lynch, M. A. (2010). The impact of glial activation in the aging brain. Aging Dis, 1(3), 262–278. 22396865 PMC3295033

[brb32736-bib-0082] Marques, F. , Sousa, J. C. , Sousa, N. , & Palha, J. A. (2013). Blood‐brain‐barriers in aging and in Alzheimer's disease. Mol Neurodegener, 8, 38. 10.1186/1750-1326-8-38 24148264 PMC4015275

[brb32736-bib-0083] Matias, I. , Morgado, J. , & Gomes, F. C. A. (2019). Astrocyte heterogeneity: Impact to brain aging and disease. Front Aging Neurosci, 11, 59. 10.3389/fnagi.2019.00059 30941031 PMC6433753

[brb32736-bib-0084] McLean, W. H. , & Lane, E. B. (1995). Intermediate filaments in disease. Curr Opin Cell Biol, 7(1), 118–125. 10.1016/0955-0674(95)80053-0 7538772

[brb32736-bib-0085] Mestre, H. , Kostrikov, S. , Mehta, R. I. , & Nedergaard, M. (2017). Perivascular spaces, glymphatic dysfunction, and small vessel disease. Clin Sci (Lond), 131(17), 2257–2274. 10.1042/CS20160381 28798076 PMC5567781

[brb32736-bib-0086] Montagne, A. , Barnes, S. R. , Sweeney, M. D. , Halliday, M. R. , Sagare, A. P. , Zhao, Z. , Toga, A. W. , Jacobs, R. E. , Liu, C. Y. , Amezcua, L. , Harrington, M. G. , Chui, H. C. , Law, M. , & Zlokovic, B. V. (2015). Blood‐brain barrier breakdown in the aging human hippocampus. Neuron, 85(2), 296–302. 10.1016/j.neuron.2014.12.032 25611508 PMC4350773

[brb32736-bib-0087] Moreno‐Jiménez, E. P. , Flor‐García, M. , Terreros‐Roncal, J. , Rábano, A. , Cafini, F. , Pallas‐Bazarra, N. , Ávila, J. , & Llorens‐Martín, M. (2019). Adult hippocampal neurogenesis is abundant in neurologically healthy subjects and drops sharply in patients with Alzheimer's disease. Nat Med, 25(4), 554–560. 10.1038/s41591-019-0375-9 30911133

[brb32736-bib-0088] Moreno‐Valladares, M. , Silva, T. M. , Garces, J. P. , Saenz‐Antonanzas, A. , Moreno‐Cugnon, L. , Alvarez‐Satta, M. , & Matheu, A. (2020). CD8(+) T cells are present at low levels in the white matter with physiological and pathological aging. Aging (Albany NY), 12(19), 18928–18941. doi:10.18632/aging.104043 33049712 PMC7732290

[brb32736-bib-0089] Morrison, J. H. , & Baxter, M. G. (2012). The ageing cortical synapse: Hallmarks and implications for cognitive decline. Nat Rev Neurosci, 13(4), 240–250. 10.1038/nrn3200 22395804 PMC3592200

[brb32736-bib-0090] Mosher, K. I. , & Wyss‐Coray, T. (2014). Microglial dysfunction in brain aging and Alzheimer's disease. Biochem Pharmacol, 88(4), 594–604. 10.1016/j.bcp.2014.01.008 24445162 PMC3972294

[brb32736-bib-0091] Mrdjen, D. , Pavlovic, A. , Hartmann, F. J. , Schreiner, B. , Utz, S. G. , Leung, B. P. , Lelios, I. , Heppner, F. L. , Kipnis, J. , Merkler, D. , Greter, M. , & Becher, B. (2018). High‐dimensional single‐cell mapping of central nervous system immune cells reveals distinct myeloid subsets in health, aging, and disease. Immunity, 48(2), 380–395. e386. 10.1016/j.immuni.2018.01.011 29426702

[brb32736-bib-0092] Murphy, G. G. , Rahnama, N. P. , & Silva, A. J. (2006). Investigation of age‐related cognitive decline using mice as a model system: behavioral correlates. Am J Geriatr Psychiatry, 14(12), 1004–1011. 10.1097/01.JGP.0000209405.27548.7b 17138807

[brb32736-bib-0093] Muzio, L. , Cavasinni, F. , Marinaro, C. , Bergamaschi, A. , Bergami, A. , Porcheri, C. , Cerri, F. , Dina, G. , Quattrini, A. , & Martino, G. (2010). Cxcl10 enhances blood cells migration in the sub‐ventricular zone of mice affected by experimental autoimmune encephalomyelitis. Mol Cell Neurosci, 43(3), 268–280. 10.1016/j.mcn.2009.11.008 19969087

[brb32736-bib-0094] Nawashiro, H. , Messing, A. , Azzam, N. , & Brenner, M. (1998). Mice lacking GFAP are hypersensitive to traumatic cerebrospinal injury. Neuroreport, 9(8), 1691–1696. 10.1097/00001756-199806010-00004 9665584

[brb32736-bib-0095] Niraula, A. , Sheridan, J. F. , & Godbout, J. P. (2017). Microglia priming with aging and stress. Neuropsychopharmacology, 42(1), 318–333. 10.1038/npp.2016.185 27604565 PMC5143497

[brb32736-bib-0096] O'Callaghan, J. P. , & Miller, D. B. (1991). The concentration of glial fibrillary acidic protein increases with age in the mouse and rat brain. Neurobiol Aging, 12(2), 171–174. 10.1016/0197-4580(91)90057-q 1904995

[brb32736-bib-0097] Ogrodnik, M. , Evans, S. A. , Fielder, E. , Victorelli, S. , Kruger, P. , Salmonowicz, H. , Weigand, B. M. , Patel, A. D. , Pirtskhalava, T. , Inman, C. L. , Johnson, K. O. , Dickinson, S. L. , Rocha, A. , Schafer, M. J. , Zhu, Y. , Allison, D. B. , Zglinicki, T. , LeBrasseur, N. K. , Tchkonia, T. , … Jurk, D. (2021). Whole‐body senescent cell clearance alleviates age‐related brain inflammation and cognitive impairment in mice. Aging Cell, 20(2), e13296. 10.1111/acel.13296 33470505 PMC7884042

[brb32736-bib-0098] Orre, M. , Kamphuis, W. , Osborn, L. M. , Melief, J. , Kooijman, L. , Huitinga, I. , Klooster, J. , Bossers, K. , & Hol, E. M. (2014). Acute isolation and transcriptome characterization of cortical astrocytes and microglia from young and aged mice. Neurobiol Aging, 35(1), 1–14. 10.1016/j.neurobiolaging.2013.07.008 23954174

[brb32736-bib-0099] Palmer, A. L. , & Ousman, S. S. (2018). Astrocytes and aging. Front Aging Neurosci, 10, 337. 10.3389/fnagi.2018.00337 30416441 PMC6212515

[brb32736-bib-0100] Pekny, M. , & Pekna, M. (2004). Astrocyte intermediate filaments in CNS pathologies and regeneration. J Pathol, 204(4), 428–437. 10.1002/path.1645 15495269

[brb32736-bib-0101] Peppiatt, C. M. , Howarth, C. , Mobbs, P. , & Attwell, D. (2006). Bidirectional control of CNS capillary diameter by pericytes. Nature, 443(7112), 700–704. 10.1038/nature05193 17036005 PMC1761848

[brb32736-bib-0102] Petri, B. , Phillipson, M. , & Kubes, P. (2008). The physiology of leukocyte recruitment: An in vivo perspective. J Immunol, 180(10), 6439–6446. 10.4049/jimmunol.180.10.6439 18453558

[brb32736-bib-0103] Pluchino, S. , Muzio, L. , Imitola, J. , Deleidi, M. , Alfaro‐Cervello, C. , Salani, G. , Porcheri, C. , Brambilla, E. , Cavasinni, F. , Bergamaschi, A. , Garcia‐Verdugo, J. M. , Comi, G. , Khoury, S. J. , & Martino, G. (2008). Persistent inflammation alters the function of the endogenous brain stem cell compartment. Brain, 131(Pt 10), 2564–2578. 10.1093/brain/awn198 18757884 PMC2570715

[brb32736-bib-0104] Propson, N. E. , Roy, E. R. , Litvinchuk, A. , Kohl, J. , & Zheng, H. (2021). Endothelial C3a receptor mediates vascular inflammation and blood‐brain barrier permeability during aging. J Clin Invest, 131(1). 10.1172/JCI140966 PMC7773352 32990682

[brb32736-bib-0105] Raber, J. , Rola, R. , LeFevour, A. , Morhardt, D. , Curley, J. , Mizumatsu, S. , VandenBerg, S. R. , & Fike, J. R. (2004). Radiation‐induced cognitive impairments are associated with changes in indicators of hippocampal neurogenesis. Radiat Res, 162(1), 39–47. 10.1667/rr3206 15222778

[brb32736-bib-0106] Reynolds, A. D. , Banerjee, R. , Liu, J. , Gendelman, H. E. , & Mosley, R. L. (2007). Neuroprotective activities of CD4+CD25+ regulatory T cells in an animal model of Parkinson's disease. J Leukoc Biol, 82(5), 1083–1094. 10.1189/jlb.0507296 17675560

[brb32736-bib-0107] Rezai‐Zadeh, K. , Gate, D. , & Town, T. (2009). CNS infiltration of peripheral immune cells: D‐Day for neurodegenerative disease? J Neuroimmune Pharmacol, 4(4), 462–475. 10.1007/s11481-009-9166-2 19669892 PMC2773117

[brb32736-bib-0108] Ritzel, R. M. , Crapser, J. , Patel, A. R. , Verma, R. , Grenier, J. M. , Chauhan, A. , Jellison, E. R. , & McCullough, L. D. (2016). Age‐associated resident memory CD8 T cells in the central nervous system are primed to potentiate inflammation after ischemic brain injury. J Immunol, 196(8), 3318–3330. 10.4049/jimmunol.1502021 26962232 PMC4868658

[brb32736-bib-0109] Rock, R. B. , Hu, S. , Deshpande, A. , Munir, S. , May, B. J. , Baker, C. A. , Peterson, P. K. , & Kapur, V. (2005). Transcriptional response of human microglial cells to interferon‐gamma. Genes Immun, 6(8), 712–719. 10.1038/sj.gene.6364246 16163375

[brb32736-bib-0110] Rodriguez, J. J. , Yeh, C. Y. , Terzieva, S. , Olabarria, M. , Kulijewicz‐Nawrot, M. , & Verkhratsky, A. (2014). Complex and region‐specific changes in astroglial markers in the aging brain. Neurobiol Aging, 35(1), 15–23. 10.1016/j.neurobiolaging.2013.07.002 23969179

[brb32736-bib-0111] Rogers, J. , Luber‐Narod, J. , Styren, S. D. , & Civin, W. H. (1988). Expression of immune system‐associated antigens by cells of the human central nervous system: relationship to the pathology of Alzheimer's disease. Neurobiol Aging, 9(4), 339–349. 10.1016/s0197-4580(88)80079-4 3263583

[brb32736-bib-0112] Rossi, B. , Angiari, S. , Zenaro, E. , Budui, S. L. , & Constantin, G. (2011). Vascular inflammation in central nervous system diseases: adhesion receptors controlling leukocyte‐endothelial interactions. J Leukoc Biol, 89(4), 539–556. 10.1189/jlb.0710432 21169520

[brb32736-bib-0113] Ruckh, J. M. , Zhao, J. W. , Shadrach, J. L. , van Wijngaarden, P. , Rao, T. N. , Wagers, A. J. , & Franklin, R. J. (2012). Rejuvenation of regeneration in the aging central nervous system. Cell Stem Cell, 10(1), 96–103. 10.1016/j.stem.2011.11.019 22226359 PMC3714794

[brb32736-bib-0114] Rustenhoven, J. , Jansson, D. , Smyth, L. C. , & Dragunow, M. (2017). Brain pericytes as mediators of neuroinflammation. Trends Pharmacol Sci, 38(3), 291–304. 10.1016/j.tips.2016.12.001 28017362

[brb32736-bib-0115] Ryman, D. , & Lamb, B. T. (2006). Genetic and environmental modifiers of Alzheimer's disease phenotypes in the mouse. Curr Alzheimer Res, 3(5), 465–473. 10.2174/156720506779025198 17168645

[brb32736-bib-0116] Salminen, A. , Kaarniranta, K. , & Kauppinen, A. (2012). Inflammaging: Disturbed interplay between autophagy and inflammasomes. Aging (Albany NY), 4(3), 166–175. 10.18632/aging.100444 22411934 PMC3348477

[brb32736-bib-0117] Saxe, M. D. , Battaglia, F. , Wang, J. W. , Malleret, G. , David, D. J. , Monckton, J. E. , Garcia, A. D. R. , Sofroniew, M. V. , Kandel, E. R. , Santarelli, L. , Hen, R. , & Drew, M. R. (2006). Ablation of hippocampal neurogenesis impairs contextual fear conditioning and synaptic plasticity in the dentate gyrus. Proc Natl Acad Sci U S A, 103(46), 17501–17506. 10.1073/pnas.0607207103 17088541 PMC1859958

[brb32736-bib-0118] Scearce‐Levie, K. (2011). Monitoring spatial learning and memory in Alzheimer's disease mouse models using the Morris Water Maze. Methods Mol Biol, 670, 191–205. 10.1007/978-1-60761-744-0_14 20967592

[brb32736-bib-0119] Schaum, N. , Lehallier, B. , Hahn, O. , Palovics, R. , Hosseinzadeh, S. , Lee, S. E. , Sit, R. , Lee, D. P. , Losada, P. M. , Zardeneta, M. E. , Fehlmann, T. , Webber, J. T. , McGeever, A. , Calcuttawala, K. , Zhang, H. , Berdnik, D. , Mathur, V. , Tan, W. , Zee, A. , … Wyss‐Coray, T. (2020). Ageing hallmarks exhibit organ‐specific temporal signatures. Nature, 583(7817), 596–602. 10.1038/s41586-020-2499-y 32669715 PMC7757734

[brb32736-bib-0120] Scheiblich, H. , Trombly, M. , Ramirez, A. , & Heneka, M. T. (2020). Neuroimmune connections in aging and neurodegenerative diseases. Trends Immunol, 10.1016/j.it.2020.02.002 32147113

[brb32736-bib-0121] Seifert, G. , Schilling, K. , & Steinhauser, C. (2006). Astrocyte dysfunction in neurological disorders: a molecular perspective. Nat Rev Neurosci, 7(3), 194–206. 10.1038/nrn1870 16495941

[brb32736-bib-0122] Shen, Q. , Zhang, R. , & Bhat, N. R. (2006). MAP kinase regulation of IP10/CXCL10 chemokine gene expression in microglial cells. Brain Res, 1086(1), 9–16. 10.1016/j.brainres.2006.02.116 16635481

[brb32736-bib-0123] Sierra, A. , Beccari, S. , Diaz‐Aparicio, I. , Encinas, J. M. , Comeau, S. , & Tremblay, M. E. (2014). Surveillance, phagocytosis, and inflammation: How never‐resting microglia influence adult hippocampal neurogenesis. Neural Plast, 2014, 610343. 10.1155/2014/610343 24772353 PMC3977558

[brb32736-bib-0124] Simard, M. , & Nedergaard, M. (2004). The neurobiology of glia in the context of water and ion homeostasis. Neuroscience, 129(4), 877–896. 10.1016/j.neuroscience.2004.09.053 15561405

[brb32736-bib-0125] Simon, M. J. , Wang, M. X. , Murchison, C. F. , Roese, N. E. , Boespflug, E. L. , Woltjer, R. L. , & Iliff, J. J. (2018). Transcriptional network analysis of human astrocytic endfoot genes reveals region‐specific associations with dementia status and tau pathology. Sci Rep, 8(1), 12389. 10.1038/s41598-018-30779-x 30120299 PMC6098119

[brb32736-bib-0126] Siracusa, R. , Fusco, R. , & Cuzzocrea, S. (2019). Astrocytes: Role and functions in brain pathologies. Front Pharmacol, 10, 1114. 10.3389/fphar.2019.01114 31611796 PMC6777416

[brb32736-bib-0127] Sofroniew, M. V. (2009). Molecular dissection of reactive astrogliosis and glial scar formation. Trends Neurosci, 32(12), 638–647. 10.1016/j.tins.2009.08.002 19782411 PMC2787735

[brb32736-bib-0128] Sofroniew, M. V. , & Vinters, H. V. (2010). Astrocytes: biology and pathology. Acta Neuropathol, 119(1), 7–35. 10.1007/s00401-009-0619-8 20012068 PMC2799634

[brb32736-bib-0129] Spencer, S. J. , D'Angelo, H. , Soch, A. , Watkins, L. R. , Maier, S. F. , & Barrientos, R. M. (2017). High‐fat diet and aging interact to produce neuroinflammation and impair hippocampal‐ and amygdalar‐dependent memory. Neurobiol Aging, 58, 88–101. 10.1016/j.neurobiolaging.2017.06.014 28719855 PMC5581696

[brb32736-bib-0130] Stichel, C. C. , & Luebbert, H. (2007). Inflammatory processes in the aging mouse brain: participation of dendritic cells and T‐cells. Neurobiol Aging, 28(10), 1507–1521. 10.1016/j.neurobiolaging.2006.07.022 16959379

[brb32736-bib-0131] Sukoff Rizzo, S. J. , Anderson, L. C. , Green, T. L. , McGarr, T. , Wells, G. , & Winter, S. S. (2018). Assessing healthspan and lifespan measures in aging mice: optimization of testing protocols, replicability, and rater reliability. Curr Protoc Mouse Biol, 8(2), e45. 10.1002/cpmo.45 29924918

[brb32736-bib-0132] Sukoff Rizzo, S. J. , & Silverman, J. L. (2016). Methodological considerations for optimizing and validating behavioral assays. Curr Protoc Mouse Biol, 6(4), 364–379. 10.1002/cpmo.17 27906464 PMC6054129

[brb32736-bib-0133] Sumbria, R. K. , Grigoryan, M. M. , Vasilevko, V. , Paganini‐Hill, A. , Kilday, K. , Kim, R. , Cribbs, D. H. , & Fisher, M. J. (2018). Aging exacerbates development of cerebral microbleeds in a mouse model. J Neuroinflammation, 15(1), 69. 10.1186/s12974-018-1092-x 29510725 PMC5840821

[brb32736-bib-0134] Sweeney, M. D. , Kisler, K. , Montagne, A. , Toga, A. W. , & Zlokovic, B. V. (2018). The role of brain vasculature in neurodegenerative disorders. Nat Neurosci, 21(10), 1318–1331. 10.1038/s41593-018-0234-x 30250261 PMC6198802

[brb32736-bib-0135] Szu, J. I. , & Binder, D. K. (2016). The role of astrocytic aquaporin‐4 in synaptic plasticity and learning and memory. Front Integr Neurosci, 10, 8. 10.3389/fnint.2016.00008 26941623 PMC4764708

[brb32736-bib-0136] Tabula Muris, C. (2020). A single‐cell transcriptomic atlas characterizes ageing tissues in the mouse. Nature, 583(7817), 590–595. 10.1038/s41586-020-2496-1 32669714 PMC8240505

[brb32736-bib-0137] Tarantini, S. , Tran, C. H. T. , Gordon, G. R. , Ungvari, Z. , & Csiszar, A. (2017). Impaired neurovascular coupling in aging and Alzheimer's disease: Contribution of astrocyte dysfunction and endothelial impairment to cognitive decline. Exp Gerontol, 94, 52–58. 10.1016/j.exger.2016.11.004 27845201 PMC5429210

[brb32736-bib-0138] Tobin, M. K. , Musaraca, K. , Disouky, A. , Shetti, A. , Bheri, A. , Honer, W. G. , Kim, N. , Dawe, R. J. , Bennett, D. A. , Arfanakis, K. , & Lazarov, O. (2019). Human hippocampal neurogenesis persists in aged adults and Alzheimer's disease patients. Cell Stem Cell, 24(6), 974–982. e973. 10.1016/j.stem.2019.05.003 31130513 PMC6608595

[brb32736-bib-0139] Togo, T. , Akiyama, H. , Iseki, E. , Kondo, H. , Ikeda, K. , Kato, M. , Oda, T. , Tsuchiya, K. , & Kosaka, K. (2002). Occurrence of T cells in the brain of Alzheimer's disease and other neurological diseases. J Neuroimmunol, 124(1‐2), 83–92. 10.1016/s0165-5728(01)00496-9 11958825

[brb32736-bib-0140] Toni, N. , & Schinder, A. F. (2015). Maturation and functional integration of new granule cells into the adult hippocampus. Cold Spring Harb Perspect Biol, 8(1), a018903. 10.1101/cshperspect.a018903 26637288 PMC4691791

[brb32736-bib-0141] Tran, T. , Mach, J. , Gemikonakli, G. , Wu, H. , Allore, H. , Howlett, S. E. , Little, C. B. , & Hilmer, S. N. (2021). Male‐female differences in the effects of age on performance measures recorded for 23 hours in mice. J Gerontol A Biol Sci Med Sci, 76(12), 2141–2146. 10.1093/gerona/glab182 34171083

[brb32736-bib-0142] Ueno, M. , Chiba, Y. , Murakami, R. , Matsumoto, K. , Fujihara, R. , Uemura, N. , Yanase, K. , & Kamada, M. (2019). Disturbance of intracerebral fluid clearance and blood‐brain barrier in vascular cognitive impairment. Int J Mol Sci, 20(10), 10.3390/ijms20102600 PMC6566824 31137875

[brb32736-bib-0143] Ungvari, Z. , Tarantini, S. , Donato, A. J. , Galvan, V. , & Csiszar, A. (2018). Mechanisms of vascular aging. Circ Res, 123(7), 849–867. 10.1161/CIRCRESAHA.118.311378 30355080 PMC6248882

[brb32736-bib-0144] van de Haar, H. J. , Burgmans, S. , Jansen, J. F. , van Osch, M. J. , van Buchem, M. A. , Muller, M. , Hofman, P. A. M. , Verhey, F. R. J. , & Backes, W. H. (2016). Blood‐brain barrier leakage in patients with early Alzheimer disease. Radiology, 281(2), 527–535. 10.1148/radiol.2016152244 27243267

[brb32736-bib-0145] Van Skike, C. E. , Jahrling, J. B. , Olson, A. B. , Sayre, N. L. , Hussong, S. A. , Ungvari, Z. , Lechleiter, J. D. , & Galvan, V. (2018). Inhibition of mTOR protects the blood‐brain barrier in models of Alzheimer's disease and vascular cognitive impairment. Am J Physiol Heart Circ Physiol, 314(4), H693–H703. 10.1152/ajpheart.00570.2017 29351469 PMC5966773

[brb32736-bib-0146] Verkhratsky, A. , Zorec, R. , Rodriguez‐Arellano, J. J. , & Parpura, V. (2019). Neuroglia in ageing. Adv Exp Med Biol, 1175, 181–197. 10.1007/978-981-13-9913-8_8 31583589 PMC7188603

[brb32736-bib-0147] Villeda, S. A. , Luo, J. , Mosher, K. I. , Zou, B. , Britschgi, M. , Bieri, G. , Stan, T. M. , Fainberg, N. , Ding, Z. , Eggel, A. , Lucin, K. M. , Czirr, E. , Park, J. ‐ S. , Couillard‐Després, S. , Aigner, L. , Li, G. , Peskind, E. R. , Kaye, J. A. , Quinn, J. F. , … Wyss‐Coray, T. (2011). The ageing systemic milieu negatively regulates neurogenesis and cognitive function. Nature, 477(7362), 90–94. 10.1038/nature10357 21886162 PMC3170097

[brb32736-bib-0148] Villeda, S. A. , Plambeck, K. E. , Middeldorp, J. , Castellano, J. M. , Mosher, K. I. , Luo, J. , Smith, L. K. , Bieri, G. , Lin, K. , Berdnik, D. , Wabl, R. , Udeochu, J. , Wheatley, E. G. , Zou, B. , Simmons, D. A. , Xie, X. S. , Longo, F. M. , & Wyss‐Coray, T. (2014). Young blood reverses age‐related impairments in cognitive function and synaptic plasticity in mice. Nat Med, 20(6), 659–663. 10.1038/nm.3569 24793238 PMC4224436

[brb32736-bib-0149] Wang, J. , Xie, L. , Yang, C. , Ren, C. , Zhou, K. , Wang, B. , Zhang, Z. , Wang, Y. , Jin, K. , & Yang, G. ‐ Y. (2015). Activated regulatory T cell regulates neural stem cell proliferation in the subventricular zone of normal and ischemic mouse brain through interleukin 10. Front Cell Neurosci, 9, 361. 10.3389/fncel.2015.00361 26441532 PMC4568339

[brb32736-bib-0150] Wang, T. , Lee, M. H. , Johnson, T. , Allie, R. , Hu, L. , Calabresi, P. A. , & Nath, A. (2010). Activated T‐cells inhibit neurogenesis by releasing granzyme B: rescue by Kv1.3 blockers. J Neurosci, 30(14), 5020–5027. 10.1523/JNEUROSCI.0311-10.2010 20371822 PMC2878660

[brb32736-bib-0151] Wang, Y. , Imitola, J. , Rasmussen, S. , O'Connor, K. C. , & Khoury, S. J. (2008). Paradoxical dysregulation of the neural stem cell pathway sonic hedgehog‐Gli1 in autoimmune encephalomyelitis and multiple sclerosis. Ann Neurol, 64(4), 417–427. 10.1002/ana.21457 18991353 PMC2757750

[brb32736-bib-0152] Weber, M. , Wu, T. , Hanson, J. E. , Alam, N. M. , Solanoy, H. , Ngu, H. , Lauffer, B. E. , Lin, H. H. , Dominguez, S. L. , Reeder, J. , Tom, J. , Steiner, P. , Foreman, O. , Prusky, G. T. , & Scearce‐Levie, K. (2015). Cognitive deficits, changes in synaptic function, and brain pathology in a mouse model of normal aging (1,2,3). eNeuro, 2(5). 10.1523/ENEURO.0047-15.2015 PMC4606159 26473169

[brb32736-bib-0153] Whitehead, J. C. , Hildebrand, B. A. , Sun, M. , Rockwood, M. R. , Rose, R. A. , Rockwood, K. , & Howlett, S. E. (2014). A clinical frailty index in aging mice: comparisons with frailty index data in humans. J Gerontol A Biol Sci Med Sci, 69(6), 621–632. 10.1093/gerona/glt136 24051346 PMC4022099

[brb32736-bib-0154] Wilcock, D. M. , Vitek, M. P. , & Colton, C. A. (2009). Vascular amyloid alters astrocytic water and potassium channels in mouse models and humans with Alzheimer's disease. Neuroscience, 159(3), 1055–1069. 10.1016/j.neuroscience.2009.01.023 19356689 PMC2699894

[brb32736-bib-0155] Williams, D. M. , Jylhava, J. , Pedersen, N. L. , & Hagg, S. (2019). A frailty index for UK biobank participants. J Gerontol A Biol Sci Med Sci, 74(4), 582–587. 10.1093/gerona/gly094 29924297 PMC6417451

[brb32736-bib-0156] Wruck, W. , & Adjaye, J. (2020). Meta‐analysis of human prefrontal cortex reveals activation of GFAP and decline of synaptic transmission in the aging brain. Acta Neuropathol Commun, 8(1), 26. 10.1186/s40478-020-00907-8 32138778 PMC7059712

[brb32736-bib-0157] Wruck, W. , Schroter, F. , & Adjaye, J. (2016). Meta‐analysis of transcriptome data related to hippocampus biopsies and iPSC‐derived neuronal cells from Alzheimer's disease patients reveals an association with FOXA1 and FOXA2 gene regulatory networks. J Alzheimers Dis, 50(4), 1065–1082. 10.3233/JAD-150733 26890743

[brb32736-bib-0158] Wu, F. , Zhao, Y. , Jiao, T. , Shi, D. , Zhu, X. , Zhang, M. , Shi, M. , & Zhou, H. (2015). CXCR2 is essential for cerebral endothelial activation and leukocyte recruitment during neuroinflammation. J Neuroinflammation, 12, 98. 10.1186/s12974-015-0316-6 25990934 PMC4438521

[brb32736-bib-0159] Wu, T. , Dejanovic, B. , Gandham, V. D. , Gogineni, A. , Edmonds, R. , Schauer, S. , Srinivasan, K. , Huntley, M. A. , Wang, Y. , Wang, T. ‐ M. , Hedehus, M. , Barck, K. H. , Stark, M. , Ngu, H. , Foreman, O. , Meilandt, W. J. , Elstrott, J. , Chang, M. C. , Hansen, D. V. , … Hanson, J. E. (2019). Complement C3 is activated in human AD brain and is required for neurodegeneration in mouse models of amyloidosis and tauopathy. Cell Rep, 28(8), 2111–2123. e2116. 10.1016/j.celrep.2019.07.060 31433986

[brb32736-bib-0160] Wyss‐Coray, T. (2016). Ageing, neurodegeneration and brain rejuvenation. Nature, 539(7628), 180–186. 10.1038/nature20411 27830812 PMC5172605

[brb32736-bib-0161] Wyss‐Coray, T. , Loike, J. D. , Brionne, T. C. , Lu, E. , Anankov, R. , Yan, F. , Silverstein, S. C. , & Husemann, J. (2003). Adult mouse astrocytes degrade amyloid‐beta in vitro and in situ. Nat Med, 9(4), 453–457. 10.1038/nm838 12612547

[brb32736-bib-0162] Xiao, Q. , Yan, P. , Ma, X. , Liu, H. , Perez, R. , Zhu, A. , Gonzales, E. , Burchett, J. M. , Schuler, D. R. , Cirrito, J. R. , Diwan, A. , & Lee, J. ‐ M. (2014). Enhancing astrocytic lysosome biogenesis facilitates Abeta clearance and attenuates amyloid plaque pathogenesis. J Neurosci, 34(29), 9607–9620. 10.1523/JNEUROSCI.3788-13.2014 25031402 PMC4099542

[brb32736-bib-0163] Xiong, X. D. , Xiong, W. D. , Xiong, S. S. , & Chen, G. H. (2018). Age‐ and gender‐based differences in nest‐building behavior and learning and memory performance measured using a radial six‐armed water maze in C57BL/6 mice. Behav Neurol, 2018, 8728415. 10.1155/2018/8728415 29854021 PMC5966705

[brb32736-bib-0164] Xu, B. , Sun, A. , He, Y. , Qian, F. , Xi, S. , Long, D. , & Chen, Y. (2018). Loss of thin spines and small synapses contributes to defective hippocampal function in aged mice. Neurobiol Aging, 71, 91–104. 10.1016/j.neurobiolaging.2018.07.010 30118927

[brb32736-bib-0165] Yang, J. , Lunde, L. K. , Nuntagij, P. , Oguchi, T. , Camassa, L. M. , Nilsson, L. N. , Lannfelt, L. , Xu, Y. , Amiry‐Moghaddam, M. , Ottersen, O. P. , & Torp, R. (2011). Loss of astrocyte polarization in the tg‐ArcSwe mouse model of Alzheimer's disease. J Alzheimers Dis, 27(4), 711–722. 10.3233/JAD-2011-110725 21891870

[brb32736-bib-0166] Yankner, B. A. , Lu, T. , & Loerch, P. (2008). The aging brain. Annu Rev Pathol, 3, 41–66. 10.1146/annurev.pathmechdis.2.010506.092044 18039130

[brb32736-bib-0167] Ye, S. M. , & Johnson, R. W. (1999). Increased interleukin‐6 expression by microglia from brain of aged mice. J Neuroimmunol, 93(1‐2), 139–148. 10.1016/s0165-5728(98)00217-3 10378877

[brb32736-bib-0168] Yemisci, M. , Gursoy‐Ozdemir, Y. , Vural, A. , Can, A. , Topalkara, K. , & Dalkara, T. (2009). Pericyte contraction induced by oxidative‐nitrative stress impairs capillary reflow despite successful opening of an occluded cerebral artery. Nat Med, 15(9), 1031–1037. 10.1038/nm.2022 19718040

[brb32736-bib-0169] Yousef, H. , Czupalla, C. J. , Lee, D. , Chen, M. B. , Burke, A. N. , Zera, K. A. , Zandstra, J. , Berber, E. , Lehallier, B. , Mathur, V. , Nair, R. V. , Bonanno, L. N. , Yang, A. C. , Peterson, T. , Hadeiba, H. , Merkel, T. , Körbelin, J. , Schwaninger, M. , Buckwalter, M. S. , … Wyss‐Coray, T. (2019). Aged blood impairs hippocampal neural precursor activity and activates microglia via brain endothelial cell VCAM1. Nat Med, 25(6), 988–1000. 10.1038/s41591-019-0440-4 31086348 PMC6642642

[brb32736-bib-0170] Yu, W. H. , Go, L. , Guinn, B. A. , Fraser, P. E. , Westaway, D. , & McLaurin, J. (2002). Phenotypic and functional changes in glial cells as a function of age. Neurobiol Aging, 23(1), 105–115. 10.1016/s0197-4580(01)00258-5 11755025

[brb32736-bib-0171] Zeppenfeld, D. M. , Simon, M. , Haswell, J. D. , D'Abreo, D. , Murchison, C. , Quinn, J. F. , Grafe, M. R. , Woltjer, R. L. , Kaye, J. , & Iliff, J. J. (2017). Association of perivascular localization of aquaporin‐4 with cognition and alzheimer disease in aging brains. JAMA Neurol, 74(1), 91–99. 10.1001/jamaneurol.2016.4370 27893874

[brb32736-bib-0172] Zhuang, J. , Zhang, L. , Dai, S. , Cui, L. , Guo, C. , Sloofman, L. , & Yang, J. (2019). Comparison of multi‐tissue aging between human and mouse. Sci Rep, 9(1), 6220. 10.1038/s41598-019-42485-3 30996271 PMC6470208

[brb32736-bib-0173] Zlokovic, B. V. (2008). The blood‐brain barrier in health and chronic neurodegenerative disorders. Neuron, 57(2), 178–201. 10.1016/j.neuron.2008.01.003 18215617

