# Supplementary figures and images for: Molecular and histological correlates of cognitive decline across age in male C57BL/6J mice
Source: Brain Behav. 2022 Aug 15;12(9):e2736. doi: 10.1002/brb3.2736 (PMC9480918; doi:10.1002/brb3.2736)

# Sup Figure 1

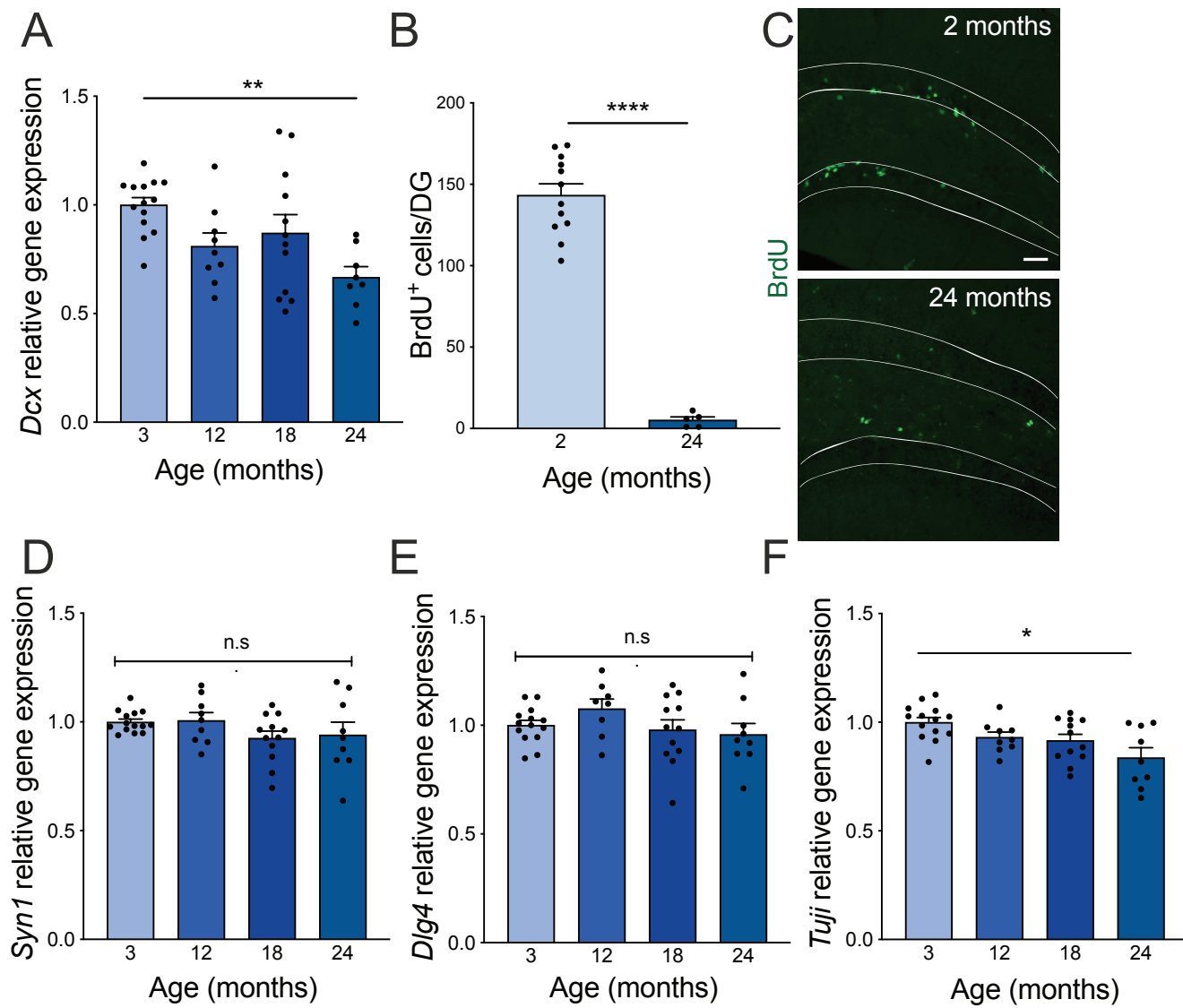

Supplement: Supplementary file 1 — Supplementary Fig. 1. Neurogenesis and proliferation are reduced in aged mice while expression of synaptic genes remains unchanged in the hippocampus. (A) Average hippocampal Dcx gene expression relative to Gapdh measured by SYBR qPCR in mice 3–24 months of age. n = 9‐14 mice per group. Kruskal‐Wallis test p = 0.0045, followed by Dunn's multiple comparisons test: 3 vs. 12 p = 0.2026, 3 vs. 18 p = 0.4264, 3 vs. 24 **p = 0.0028, 12 vs. 18 p>0.9999, 12 vs. 24 p>0.9999, 18 vs. 24 p = 0.3888. (B) Young (2 month) and aged (24 month) mice were dosed with saturating amounts of BrdU IP (2 months ‐ 500mg/kg; 24 months ‐ 150mg/kg) daily for 7 days to label proliferating cells then sacrificed 24 hours following the final injection to assess the number of BrdU‐positive cells per dentate gyrus (DG). n = 5‐12 mice per group. Nested t‐test F = 156.9, ****p<0.0001. (C) Representative images of BrdU staining (green) in the DG (outlined) of young (2 month) and aged (24 month) mice. Scale bar 100mm. (D) Average hippocampal Syn1 gene expression relative to Gapdh measured by SYBR qPCR in mice 3–24 months of age. n = 9‐14 mice per group. Kruskal‐Wallis test p = 0.2266. (E) Average hippocampal Dlg4 gene expression relative to Gapdh measured by SYBR qPCR in mice 3–24 months of age. n = 8‐14 mice per group. Kruskal‐Wallis test p = 0.2879. (F) Average hippocampal Tuj1 gene expression relative to Gapdh measured by SYBR qPCR in mice 3–24 months of age. n = 9‐14 mice per group. Kruskal‐Wallis test p = 0.0161, followed by Dunn's multiple comparisons test: 3 vs. 12 p = 0.4602, 3 vs. 18 p = 0.2752, 3 vs. 24 *p = 0.0118, 12 vs. 18 p>0.9999, 12 vs. 24 p>0.9999, 18 vs. 24 p>0.9999. All data are shown as mean ± s.e.m. Abbreviations: Dcx, doublecortin; Gapdh, glyceraldehyde‐3‐phosphate dehydrogenase; qPCR, quantitative polymerase chain reaction; BrdU, 5‐bromo‐2’‐deoxyuridine; IP, intraperitoneal; DG, dentate gyrus; Syn1, synapsin 1; Dlg4, discs large MAGUK scaffold protein 4; Tuj1, tubulin beta 3 class [file BRB3-12-e2736-s002.pdf]

Sup Figure 2

A

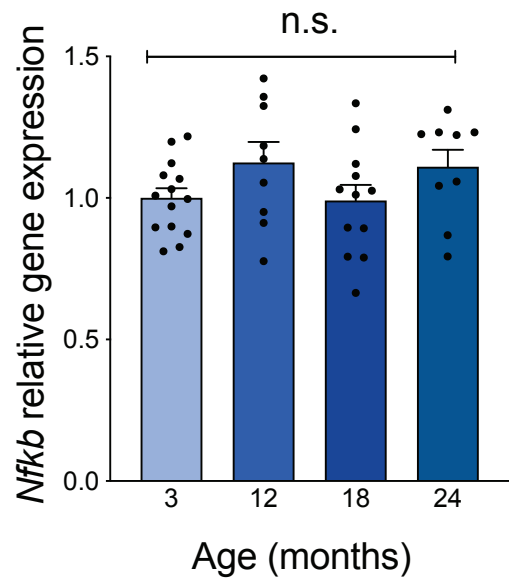

B

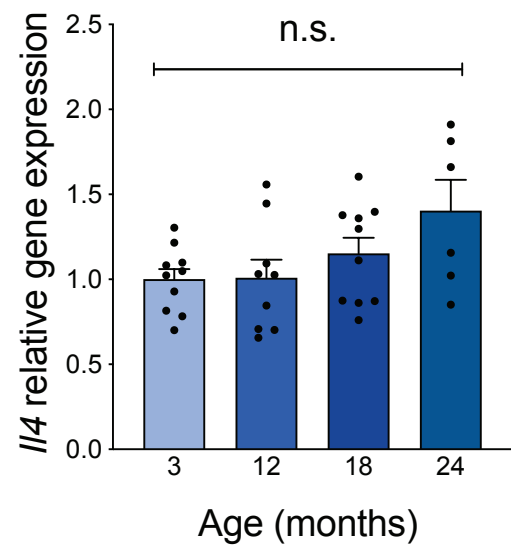

C

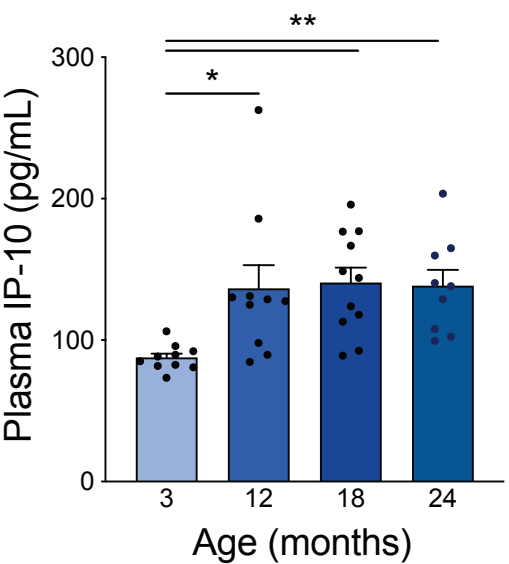

D

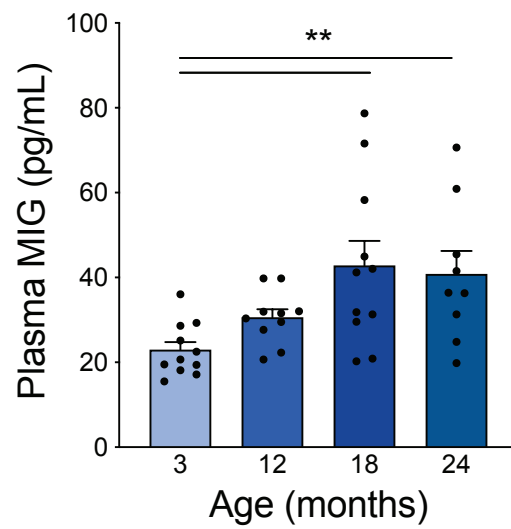

Supplement: Supplementary file 2 — Supplementary Fig. 2. Proinflammatory cytokines are elevated with age while RNA expression of some microglial genes from bulk hippocampal tissue are unchanged. (A) Average hippocampal Nfkb gene expression relative to Gapdh measured by Taqman qPCR in mice 3–24 months of age. n = 9‐14 mice per group. Kruskal‐Wallis test p = 0.2192. (B)Average hippocampal Il4 gene expression relative to Gapdh measured by SYBR qPCR in mice 3–24 months of age. n = 6‐10 mice per group. Kruskal‐Wallis test p = 0.1836. (C) Average circulating plasma levels of IP‐10 (pg/mL) measured by Luminex in mice 3–24 months of age. n = 9‐11 mice per group. Kruskal‐Wallis test p = 0.0006, followed by Dunn's multiple comparisons test: 3 vs. 12 *p = 0.0182, 3 vs. 18 **p = 0.0017, 3 vs. 24 ***p = 0.0028, 12 vs. 18 p>0.9999, 12 vs. 24 p>0.9999, 18 vs. 24 p>0.9999. (D) Average circulating plasma levels of MIG (pg/mL) measured by Luminex in mice 3–24 months of age. n = 9‐11 mice per group. Kruskal‐Wallis test p = 0.0021, followed by Dunn's multiple comparisons test: 3 vs. 12 p = 0.2348, 3 vs. 18 **p = 0.0040, 3 vs. 24 **p = 0.0089, 12 vs. 18 p>0.9999, 12 vs. 24 p>0.9999, 18 vs. 24 p>0.9999. All data are shown as mean ± s.e.m. Abbreviations: RNA, ribonucleic acid; Nfkb, Nuclear factor kappa beta subunit; Gapdh, glyceraldehyde‐3‐phosphate dehydrogenase; qPCR, quantitative polymerase chain reaction; Il4, interleukin 4; IP‐10, interferon gamma‐induced protein 10; MIG, monokine induced by gamma interferon. [file BRB3-12-e2736-s001.pdf]

# Sup Figure 3

A

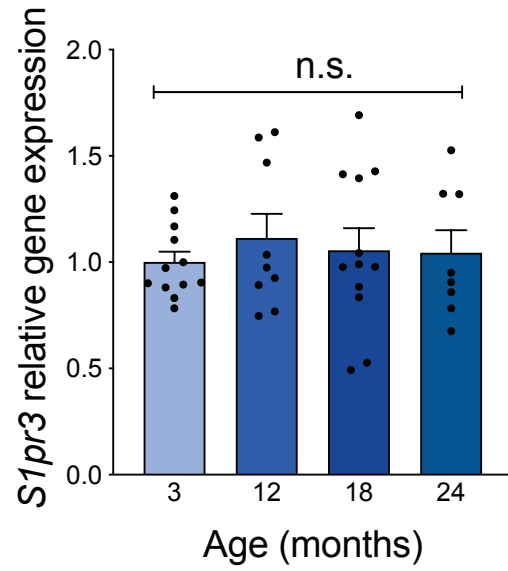

B

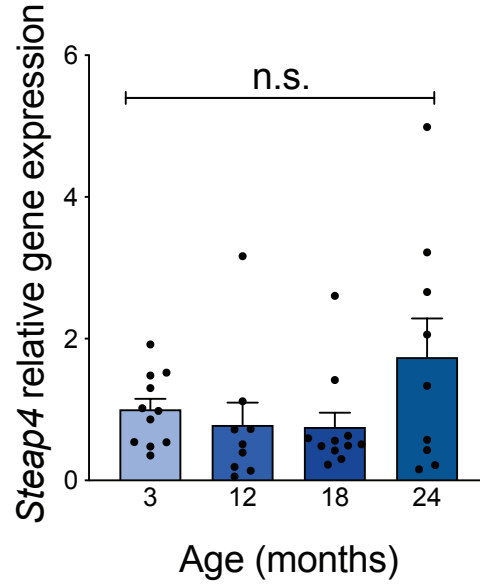

C

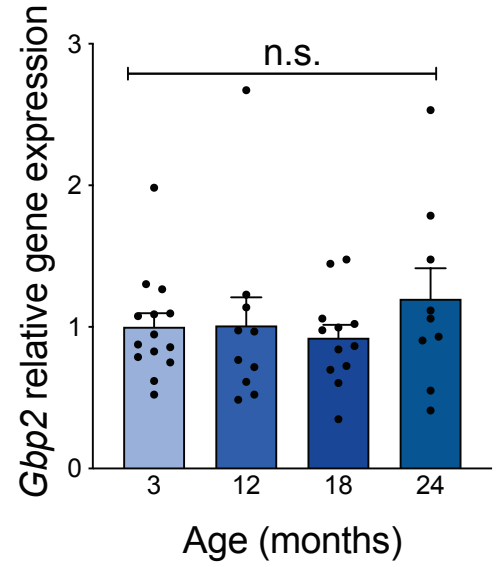

D

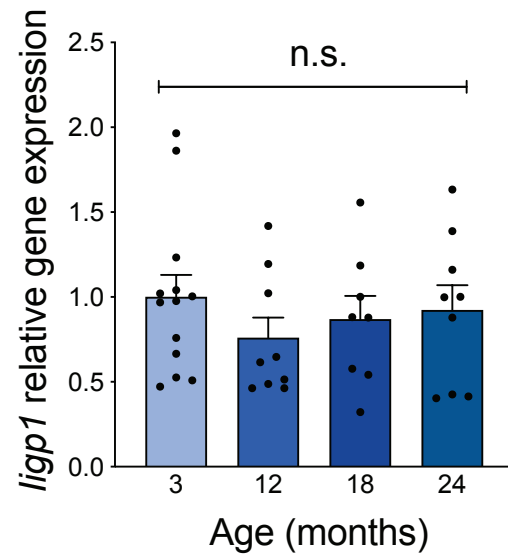

E

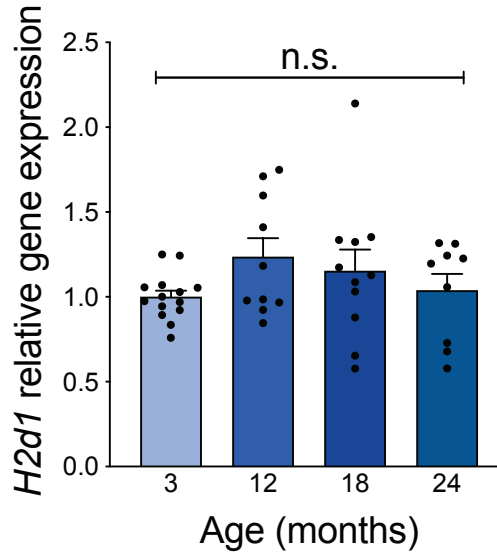

F

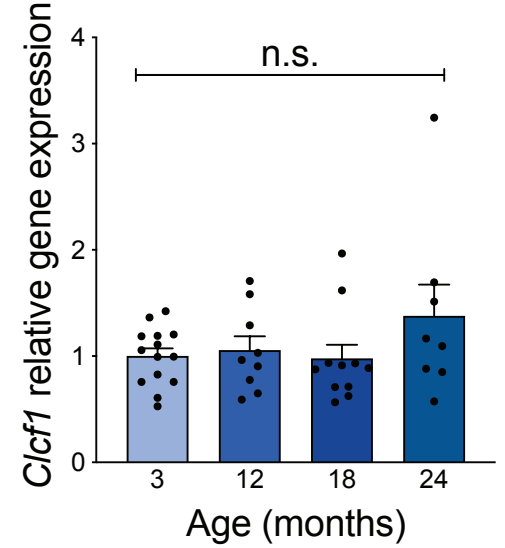

Supplement: Supplementary file 3 — Supplementary Fig. 3. Expression of astrocytic reactivity genes from bulk hippocampal tissue are unchanged with age. (A) Average hippocampal S1pr3 gene expression relative to Gapdh measured by SYBR qPCR in mice 3–24 months of age. n = 8‐12 mice per group. Kruskal‐Wallis test p = 0.9098. (B) Average hippocampal Steap4 gene expression relative to Gapdh measured by SYBR qPCR in mice 3–24 months of age. n = 9‐11 mice per group. Kruskal‐Wallis test p = 0.2849. (C) Average hippocampal Gbp2 gene expression relative to Gapdh measured by SYBR qPCR in mice 3–24 months of age. n = 9‐14 mice per group. Kruskal‐Wallis test p = 0.7172. (D) Average hippocampal Iigp1 gene expression relative to Gapdh measured by SYBR qPCR in mice 3–24 months of age. n = 8‐13 mice per group. Kruskal‐Wallis test p = 0.7109. (E) Average hippocampal H2d1 gene expression relative to Gapdh measured by SYBR qPCR in mice 3–24 months of age. n = 9‐14 mice per group. Kruskal‐Wallis test p = 0.3997. (F) Average hippocampal Clcf1 gene expression relative to Gapdh measured by SYBR qPCR in mice 3–24 months of age. n = 8‐14 mice per group. Kruskal‐Wallis test p = 0.5791. All data are shown as mean ± s.e.m. Abbreviations: S1pr3, sphingosine‐1‐phosphate receptor 3; Gapdh, glyceraldehyde‐3‐phosphate dehydrogenase; qPCR, quantitative polymerase chain reaction; Steap4, six transmembrane epithelial antigen of prostate 4; Gbp2, guanylate binding protein 2; Iigp1, interferon‐gamma‐inducible GTPase Ifgga1 protein; H2d1, histocompatibility 2 D region locus 1; Clcf1, cardiotrophin Like Cytokine Factor 1. [file BRB3-12-e2736-s003.pdf]
